# Supplementary material for: Pantothenate biosynthesis is critical for chronic infection by the neurotropic parasite Toxoplasma gondii
Source: Nat Commun. 2022 Jan 17;13:345. doi: 10.1038/s41467-022-27996-4 (PMC8764084; doi:10.1038/s41467-022-27996-4)
Supplement: Supplementary file 1 — Supplementary Information [file 41467_2022_27996_MOESM1_ESM.pdf]

Supplementary Information File

Pantothenate biosynthesis is critical for chronic infection by the neurotropic parasite *Toxoplasma gondii*

Matteo Lunghi*, Joachim Kloehn*, Aarti Krishnan, Emmanuel Varesio, Oscar Vadas, and Dominique Soldati-Favre#

* These authors contributed equally to the work

# Corresponding author: Dominique.Soldati-Favre@unige.ch

This pdf file includes:

- Supplementary Figures 1 to 8
- Supplementary Table 1
- Supplementary Methods
- Raw blots and gels of Supplementary Figures

Supplementary Figures

Supplementary Fig. 1

a Cartoons representations and integration PCRs of PanK1 and PanK2 mAID-HA tagging constructs in Tir1 parasites. Expected base pairs, as well as numbered primers are indicated. **b** Western blot of IAA treated (hours indicated) PanK1-mAID-HA/PanK2-Ty strain (n = 3) or PanK2-mAID-HA/PanK1-Ty (n = 3), respectively. Antibodies anti HA and anti Ty for the detection of the tagged proteins, anti actin as loading control. **c** Cartoon representation and integration PCR for the PPCDC-mAID-HA tagging construct in Tir1 parasite. Expected base pairs, as well as numbered primers are indicated **d** Plaque assay of PPCDC-mAID-HA parasites in presence of IAA, with the supplementation of 250 µM, 500 µM. and 1 mM Pan, pantetheine (PanSH), or CoA (n =3). **e** Cartoon representation and integration PCR for the generation of DPCK-iKD parasites. Expected base pairs, as well as numbered primers are indicated. n number of independent biological replicates. Black asterisks indicate unspecific signal, red asterisks indicate degradation products of the tagged protein. 1Kb plus DNA ladder (Thermo 10787018) was used for all agarose gel electrophoresis. Black scale bar 1 mm. Source data are provided in the Supplementary File.

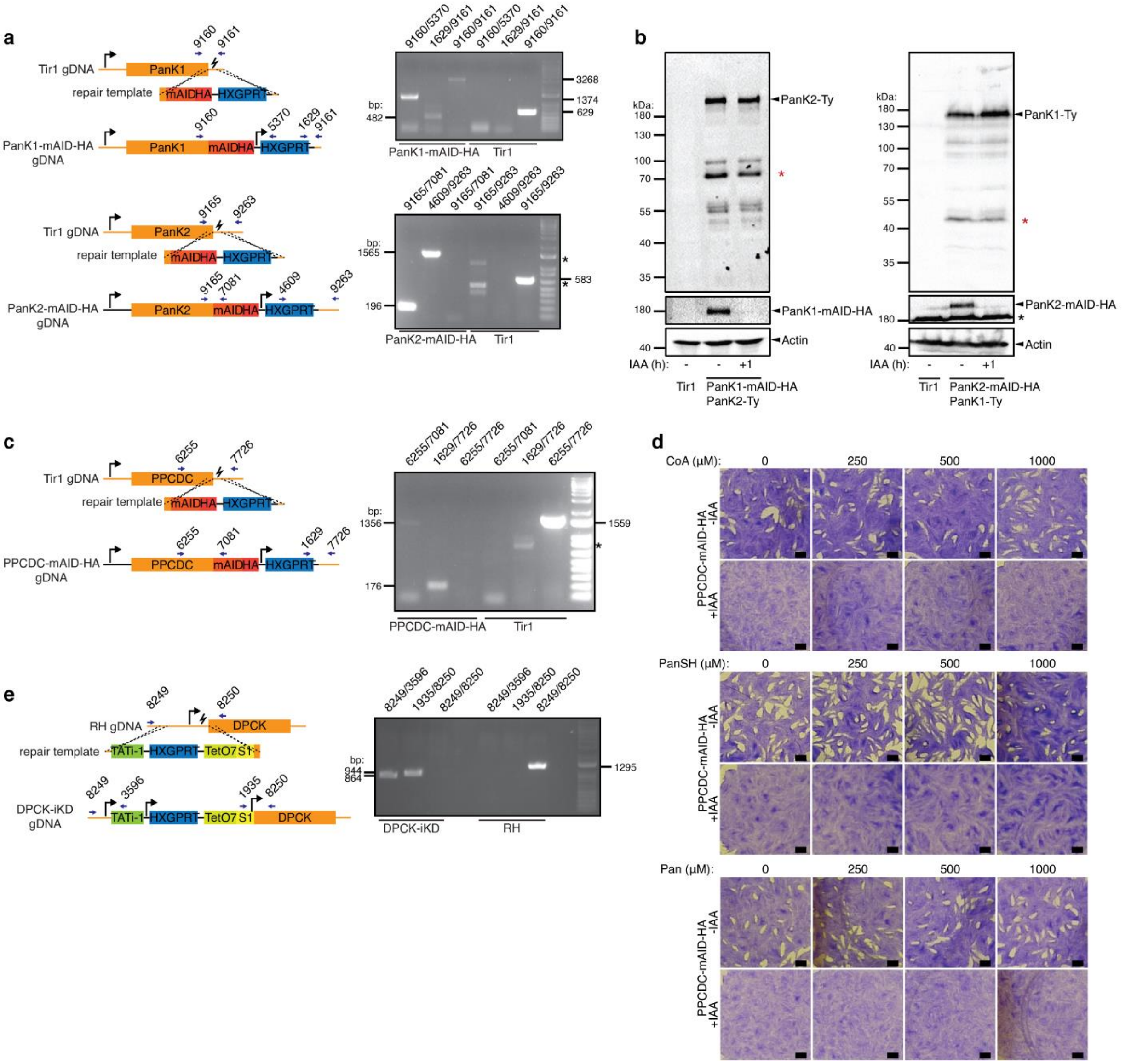

a
Tir1 gDNA
PanK1
9160
9161
repair template
mAIDHA
HXGPRT
PanK1-mAID-HA gDNA
5370
1629
9160/5370
1629/9161
9160/9161
bp:
482
3268
1374
629
PanK1-mAID-HA
Tir1
PanK2
9165
9263
PanK2-mAID-HA gDNA
7081
4609
9165/7081
4609/9263
9165/9263
1565
196
583
PanK2-mAID-HA
Tir1
b
kDa:
180
130
100
70
55
40
35
PanK2-Ty
PanK1-mAID-HA
Actin
IAA (h):
-
+1
Tir1
PanK1-mAID-HA PanK2-Ty
PanK1-Ty
PanK2-mAID-HA
PanK2-mAID-HA PanK1-Ty
c
Tir1 gDNA
PPCDC
6255
7726
repair template
mAIDHA
HXGPRT
PPCDC-mAID-HA gDNA
7081
1629
6255/7081
1629/7726
6255/7726
bp:
1356
176
1559
PPCDC-mAID-HA
Tir1
d
CoA (μM):
0
250
500
1000
PPCDC-mAID-HA -IAA
PPCDC-mAID-HA +IAA
PanSH (μM):
Pan (μM):
e
RH gDNA
8249
8250
DPCK
repair template
TATi-1
HXGPRT
TetO7S1
DPCK-iKD gDNA
3596
1935
8249/3596
1935/8250
8249/8250
bp:
944
864
1295
DPCK-iKD
RH

Supplementary Fig. 2

a Cartoon representation and integration PCR for the knockout of *kphmt-kpr* and *pbal* genes, respectively, in RH parasites. Expected base pairs, as well as numbered primers are indicated, asterisks mark unspecific bands. **b** Fractional isotopologue distribution of a Pan fragment by GC-MS analysis (*m/z* 201) in parasites cultured in regular medium or in medium –Pan supplemented with labeled $^{13}C_5/^{15}N$-Pan following correction for natural abundance (n = 3, mean and SD). 1Kb plus DNA ladder (Thermo 10787018) was used for all agarose gel electrophoresis.). n number of independent biological replicates. Source data are provided in the Supplementary File.

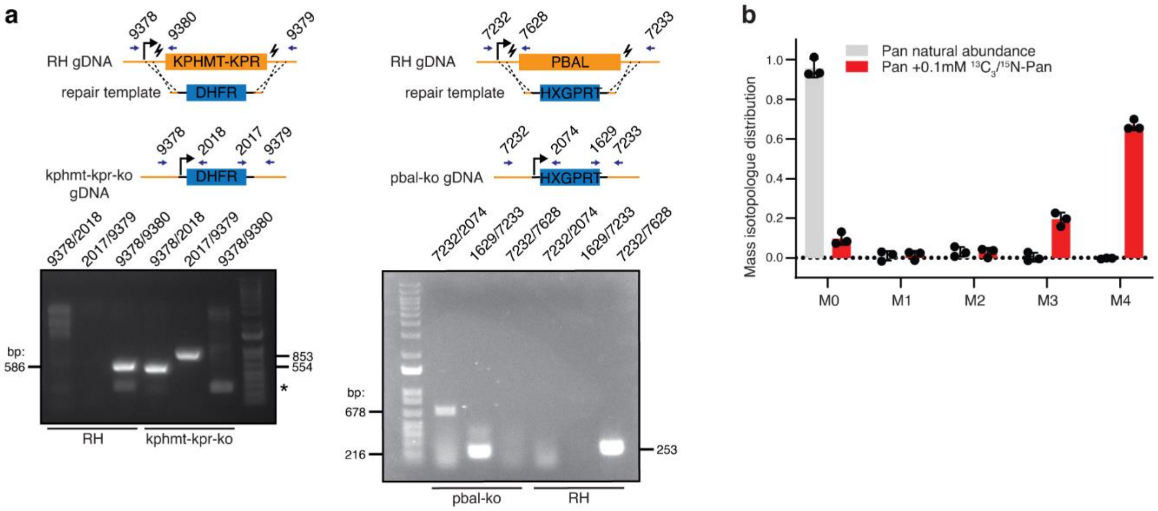

Supplementary Fig. 3

GC-MS total ion chromatogram (TIC) of polar metabolites extracted from standard DMEM (upper panel), and custom-made Pan depleted DMEM, with dialyzed FBS (lower panel). Peak corresponding to Pan is highlighted and the selected ion chromatograms of its qualifier ions (*m/z* 201, 291 and 420) are displayed in the insert panel. No TIC peaks or ions corresponding to Pan were detected in the –Pan medium (lower panel).

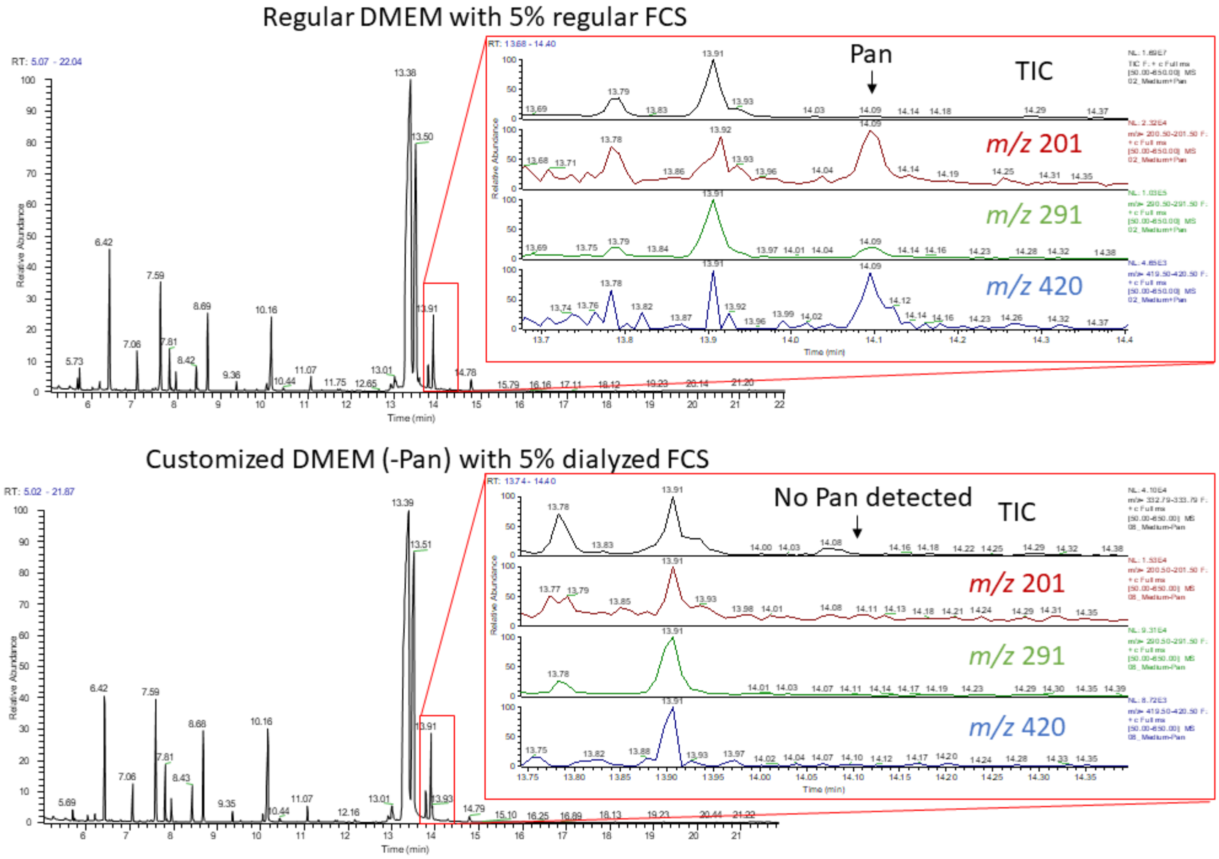

Supplementary Fig. 4

a GC-MS total ion chromatogram of polar metabolites extracted from *T. gondii* tachyzoites cultured under standard conditions (DMEM supplemented with 5% FBS). Peaks corresponding to three metabolites of interest are highlighted and their mass spectra are displayed: 2-trimethylsilyl-(TMS)-valine (Val), 3-TMS-β-alanine (β-Ala), and 3-TMS-pantothenate (Pan). Qualifier ions are highlighted in red. All metabolites were identified based on the retention time and ion chromatogram of authentic standards (natural abundance as well as stable isotope labeled metabolites). **b** Representative LC-MS total ion chromatogram (TIC) of parasites grown under standard culture conditions and selected ion chromatograms corresponding to the mass of l-alanine, β-Ala and Pan $[M+H]^{+}$-ions. The accurate mass, retention time and peak intensity are given. Metabolite extracts were analyzed in positive mode and metabolites were identified based on the retention time and accurate mass of authentic standards.

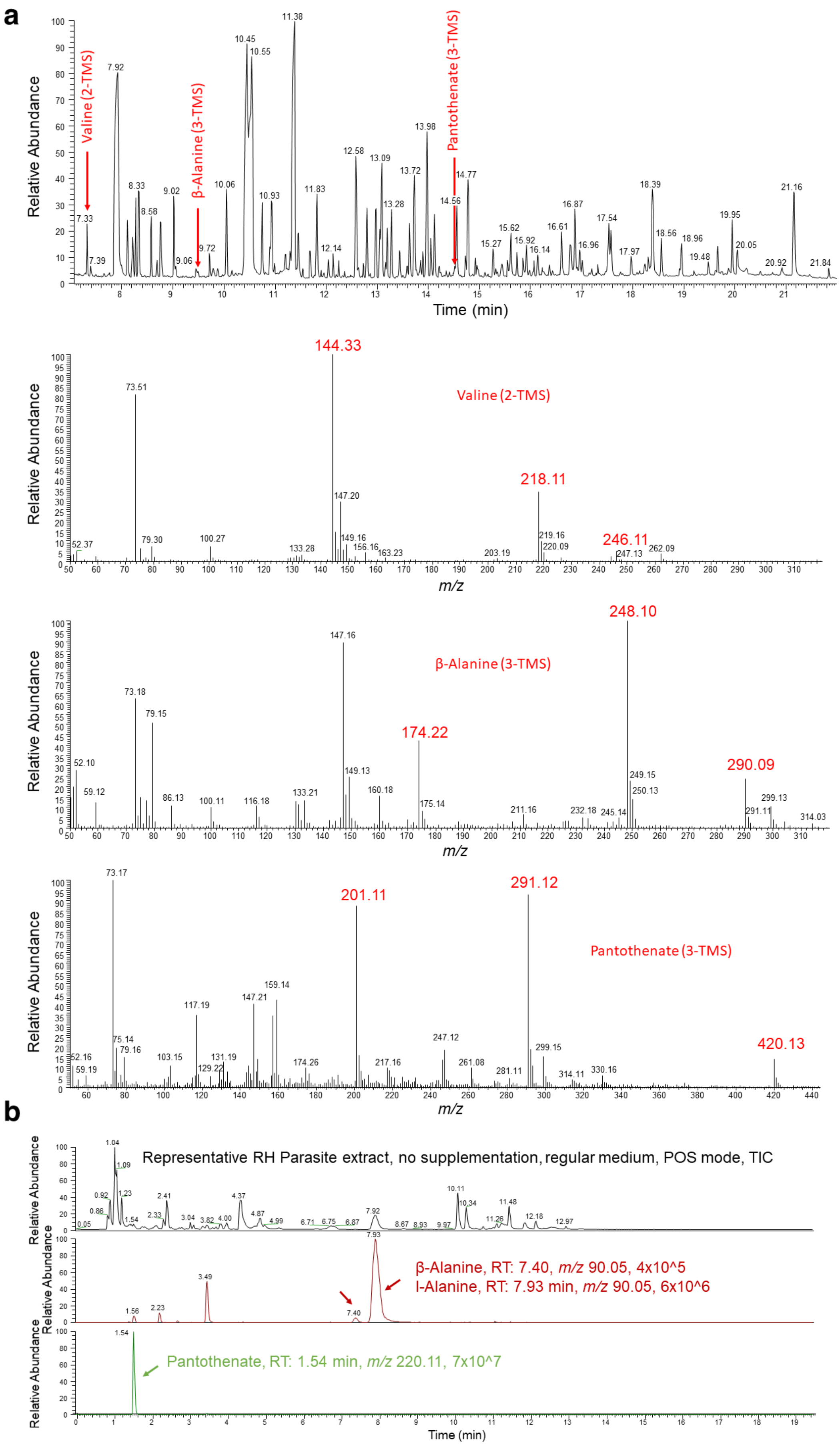
a
Relative Abundance
Time (min)
Valine (2-TMS)
β-Alanine (3-TMS)
Pantothenate (3-TMS)
m/z
144.33
218.11
246.11
248.10
174.22
290.09
201.11
291.12
420.13
b
Representative RH Parasite extract, no supplementation, regular medium, POS mode, TIC
β-Alanine, RT: 7.40, m/z 90.05, 4x10^5
l-Alanine, RT: 7.93 min, m/z 90.05, 6x10^6
Pantothenate, RT: 1.54 min, m/z 220.11, 7x10^7

Supplementary Fig. 5

Representative LC-MS total ion chromatogram (TIC) of a cell extract of parasites grown in medium containing 0.8 mM $^{13}C_3/^{15}N$-β-Ala and selected ion chromatograms corresponding to the mass of unlabeled l-alanine, β-Ala and $^{13}C_3/^{15}N$-β-Ala $[M+H]^+$-ions. The accurate mass, retention time and peak intensity are given. Metabolite extracts were analyzed in positive mode and metabolites were identified based on the retention time and accurate mass of authentic standards.

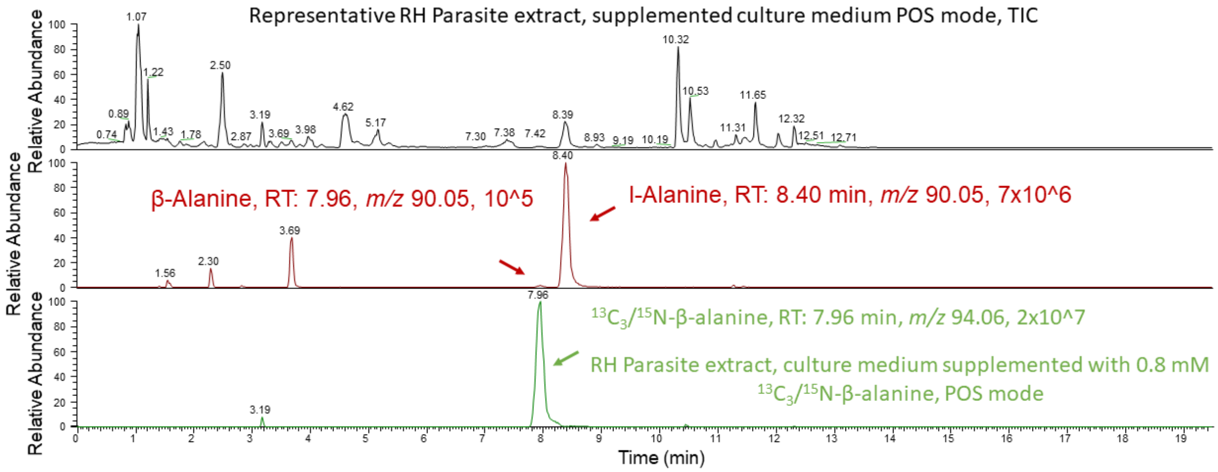

Supplementary Fig. 6

Size exclusion chromatography of *E. coli* purified MBP-PBALcd. In the inset, coomassie-stained gel of the purified MBP-PBALcd (expected MW of 92 kDa). Source data are provided in the Supplementary File.

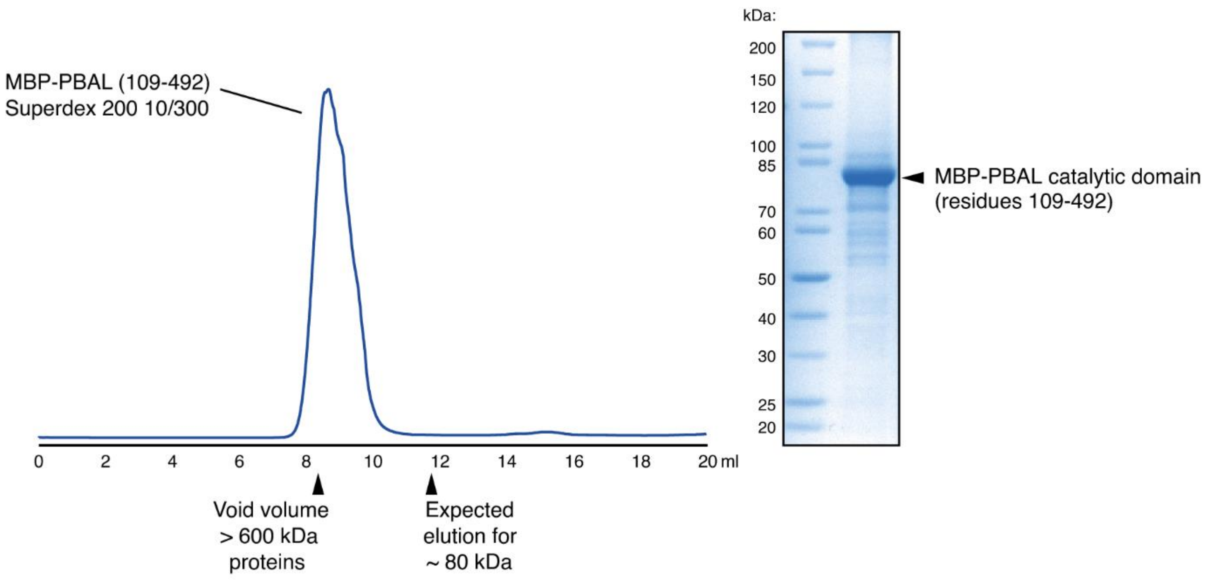

Supplementary Fig. 7

a Upper panels: LC-MS total ion chromatogram of a standard metabolite mix containing Pan, α-ketoisovalerate, pantoate, β-Ala and l-alanine and overlayed ion chromatograms corresponding to the $[M-H]^-$ ions of Pan, pantoate and α-ketoisovalerate detected in negative mode. The retention time and accurate mass are given. Lower panels: Standard metabolite mix as above but analyzed in positive mode. The selected ion chromatograms corresponding to the mass of unlabeled l-alanine, β-Ala and $^{13}C_3$/^{15}N-β-Ala $[M+H]^+$-ions are displayed in the bottom panel. The retention time and accurate mass are given. **b** Representative LC-MS total ion chromatogram of a parasite extract of cells grown under standard culture conditions. The selected ion chromatograms corresponding to the $[M-H]^-$-ions of Pan, pantoate and α-ketoisovalerate in negative mode are shown. Pan is abundantly detected, while pantoate is absent and α-ketoisovalerate is just detected above background noise. **c** Representative LC-MS total ion chromatogram (TIC, upper panel) of a parasite extract of cells grown in medium supplemented with 0.8 mM α-ketoisovalerate (middle panel) or 0.8 mM pantoate (bottom panel). The selected ion chromatograms corresponding to the $[M-H]^-$-ions of pantoate and α-ketoisovalerate detected in negative mode are shown. The accurate mass, retention time and peak intensity are given.

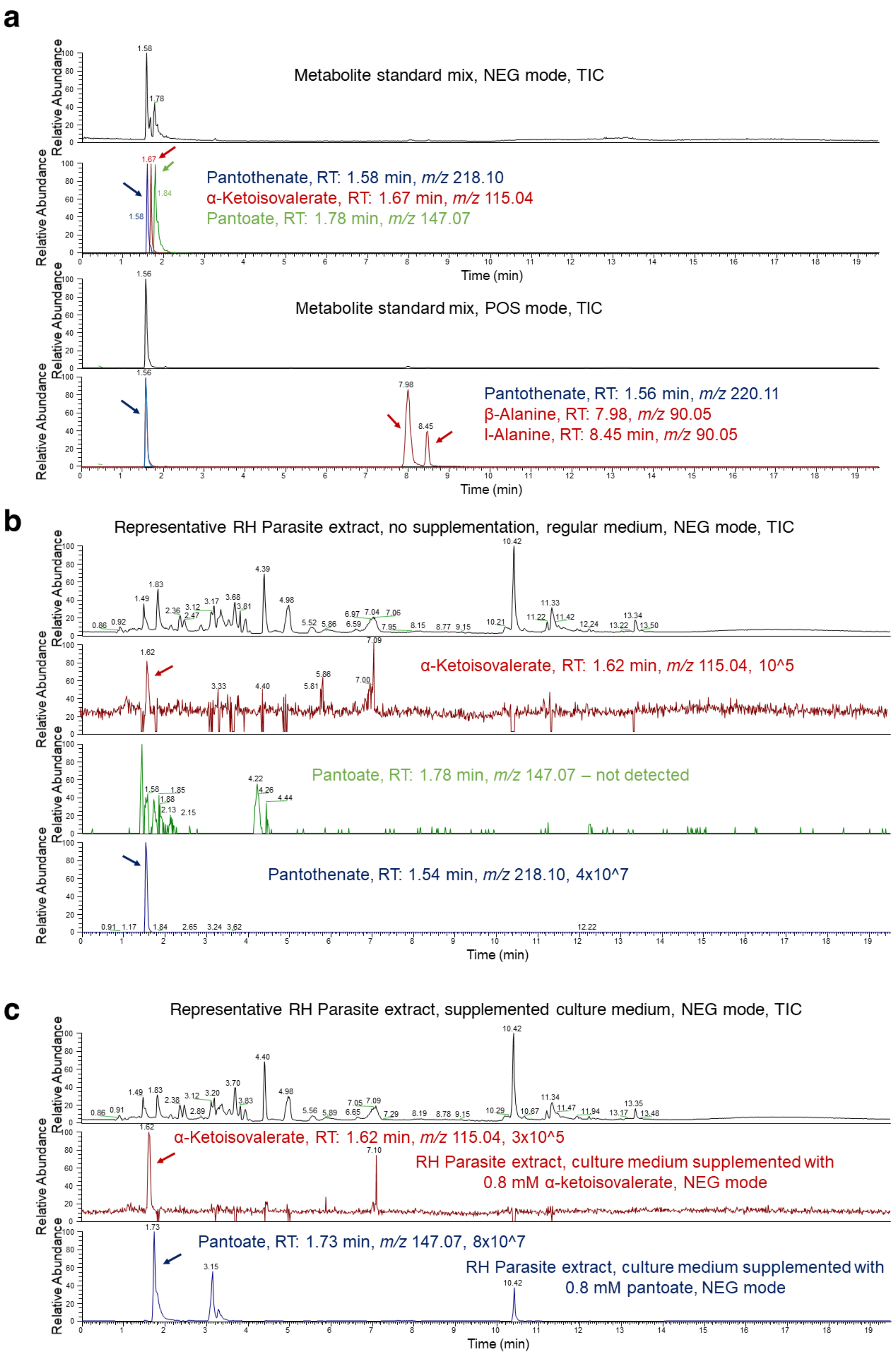

a
Metabolite standard mix, NEG mode, TIC
Pantothenate, RT: 1.58 min, m/z 218.10
α-Ketoisovalerate, RT: 1.67 min, m/z 115.04
Pantoate, RT: 1.78 min, m/z 147.07
Metabolite standard mix, POS mode, TIC
Pantothenate, RT: 1.56 min, m/z 220.11
β-Alanine, RT: 7.98, m/z 90.05
l-Alanine, RT: 8.45 min, m/z 90.05
Relative Abundance
Time (min)
b
Representative RH Parasite extract, no supplementation, regular medium, NEG mode, TIC
α-Ketoisovalerate, RT: 1.62 min, m/z 115.04, 10^5
Pantoate, RT: 1.78 min, m/z 147.07 – not detected
Pantothenate, RT: 1.54 min, m/z 218.10, 4x10^7
c
Representative RH Parasite extract, supplemented culture medium, NEG mode, TIC
α-Ketoisovalerate, RT: 1.62 min, m/z 115.04, 3x10^5
RH Parasite extract, culture medium supplemented with
0.8 mM α-ketoisovalerate, NEG mode
Pantoate, RT: 1.73 min, m/z 147.07, 8x10^7
RH Parasite extract, culture medium supplemented with
0.8 mM pantoate, NEG mode

Supplementary Fig. 8

a Percent labeling of Pan when RH parental, *bcat-ko*, *kphmt-kpr-ko*, and *pbal-ko* were grown in medium supplemented with $^{13}C_3/^{15}N$-β-Ala, and pantotate or α-ketoisovalerate (all 0.8 mM). The percent labeling was calculated based on the abundance of all detected isotopologues of Pan, and correction of natural abundance background (negative mode analysis, n = 3, mean and SD). **b** Percent labeling of Pan when ME49 parental tachyzoites and 6 days alkaline-stressed bradyzoites were grown in medium supplemented with $^{13}C_3/^{15}N$-β-Ala, and pantotate or α-ketoisovalerate (all 0.8 mM). The percent labeling was calculated based on the abundance of all detected isotopologues of Pan, and correction of natural abundance background (negative mode analysis, n = 3, mean and SD). **c** GC-MS analysis of Pan labeling in ME49 parental tachyzoite metabolite extracts of parasites incubated for 40 hours in medium containing 2 mM $^{13}C_3/^{15}N$-β-Ala. The percent labeling was calculated based on the abundance of all detected isotopologues of Pan, and correction of natural abundance background (n = 3, mean and SD). **d** Percent labeling of Pan from parasite extracts when ME49 parental tachyzoites and 6 days Shield treated ddBFD1 bradyzoites were grown in medium supplemented with $^{13}C_3/^{15}N$-β-Ala (0.8 mM). The percent labeling was calculated based on the abundance of all detected isotopologues of Pan, and correction of natural abundance background (n = 4, mean and SD). n number of independent biological replicates. Source data are provided in the Supplementary File.

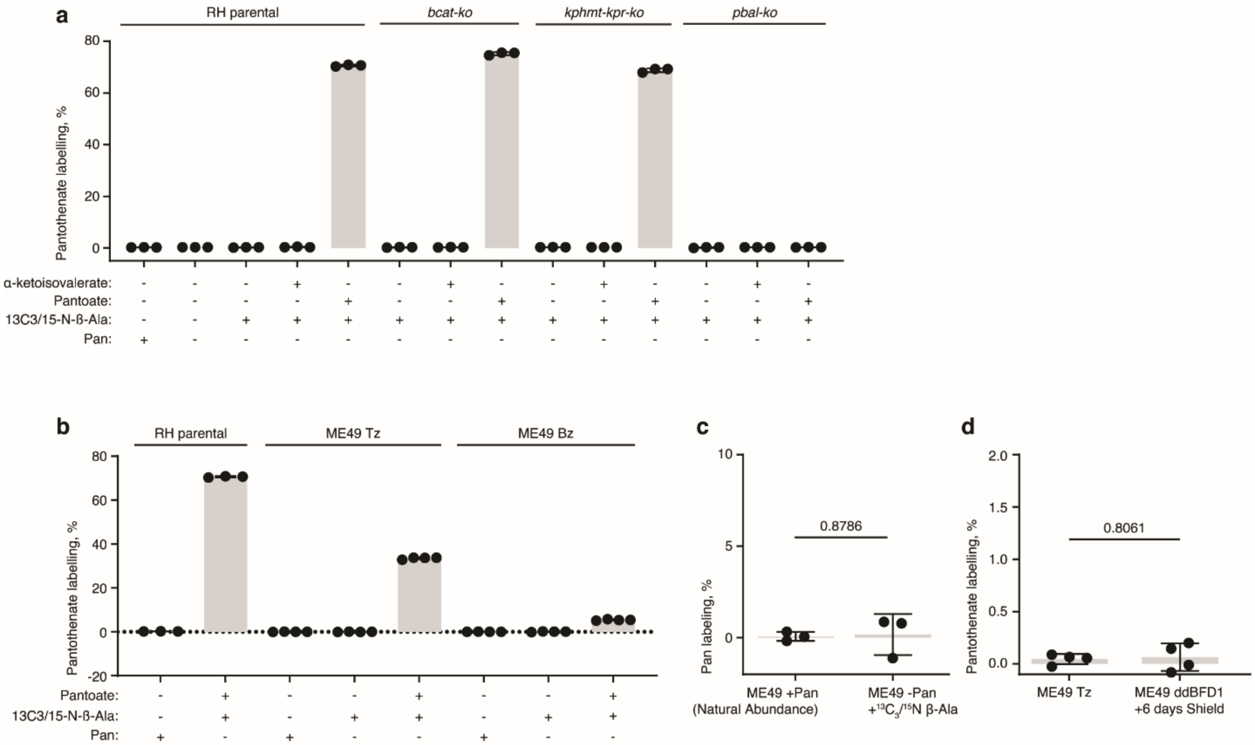

a
RH parental
bcat-ko
kphmt-kpr-ko
pbal-ko
Pantothenate labelling, %
α-ketoisovalerate:
Pantoate:
13C3/15-N-β-Ala:
Pan:
b
RH parental
ME49 Tz
ME49 Bz
Pantothenate labelling, %
Pantoate:
13C3/15-N-β-Ala:
Pan:
c
0.8786
Pan labeling, %
ME49 +Pan
(Natural Abundance)
ME49 -Pan
+13C3/15N β-Ala
d
0.8061
Pantothenate labelling, %
ME49 Tz
ME49 ddBFD1
+6 days Shield

Supplementary Fig. 9

a Cartoon representation and integration PCR for the knockout of *kphmt-kpr* and *pbal* genes, respectively, in ME49 parasites. Expected base pairs, as well as numbered primers are indicated. **b** Cartoon representation and integration PCR for the knock-in of ::c*Tg*PBAL-myc and c*Ec*PBAL-myc in the *UPRT* locus for complementation of ME49 *pbal-ko*, respectively. Expected base pairs, as well as numbered primers are indicated. **c** Western blot of seroconversion of *pbal-ko* infected mice that did not present any brain cysts (1 mouse from all experiments). 1Kb plus DNA ladder (Thermo 10787018) was used for all agarose gel electrophoresis. Source data are provided in the Supplementary File.

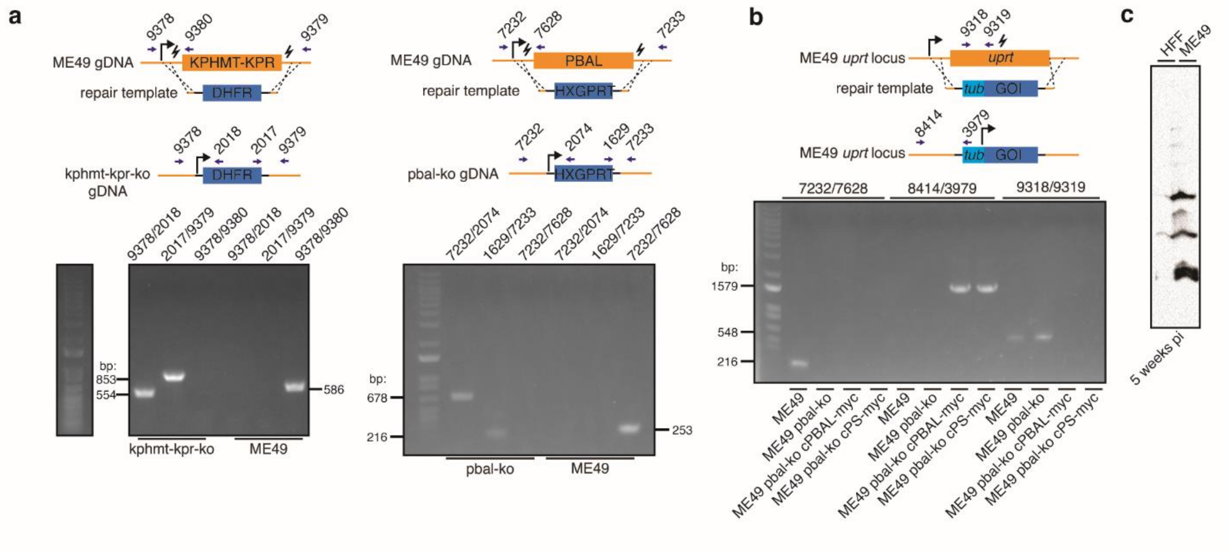

Supplementary Table 1 – Primers

construct	number	sequence	description
PanK1-mAID-HA	9157	GTCTCGAATGGACTCGAATGAGTTTTAGAGCTAGAAATAGC	gRNA cloning
	9158	GACGAATGCCCCGTGGAAAAATCCCGCTGCGCTAGCAAGGGCTCGGG	PCR homology template
	9159	ATGGATACAATGCGGCTAAAGACAACGGCCTGGAGCTCCACCGCGG	PCR homology template
	9160	GGCGCTTGGAGCTCTGG	5' integration forward
	9161	ACGCATTTGCCTCGATCC	3' integration reverse
PanK1-Ty	9266	GACGAATGCCCCGTGGAAAAATCCCGCTGCCGCCCCGCGCGCGATGCA	PCR homology template
	9267	ATGGATACAATGCGGCTAAAGACAACGGCCGCTGGAGCTCCACCGCGG	PCR homology template
PanK2-mAID-HA	9162	GAGGTGGAAAGGCGTGCAGCGGTTTTAGAGCTAGAAATAGC	gRNA cloning
	9163	CACGTTCTTCCTGCCAGAGAAGAGAGCGAGGCTAGCAAGGGCTCGGG	PCR homology template
	9164	TCTGTGTCTCTCGAGAGCACTCCGGGCTCCTGGAGCTCCACCGCGG	PCR homology template
	9165	CACTCCTCGCCGTACGC	5' integration forward
	9263	CTCTGTGTCGACGTACTGCC	3' integration reverse
PanK2-Ty	9268	CACGTTCTTCCTGCCAGAGAAGAGAGCGAGCGCCCCGCGCGCGATGCA	PCR homology template
	9269	TCTGTGTCTCTCGAGAGCACTCCGGGCTCCGCTGGAGCTCCACCGCGG	PCR homology template
PPCDC-mAID-HA	7897	GACAAGTAGGCAAGAGCAGTGGTTTTAGAGCTAGAAATAGC	gRNA cloning
	7798	TCTGCGTGCTCTATGCAGACACAAAGGTTCGCTAGCAAGGGCTCGGG	PCR homology template
	7799	ACATTGAAGACCTGTGATGGAAGAGAAGAGGAATTGGAGCTCCACCGC	PCR homology template
	6255	GAAAGAGATGCGAGTACAGAGAG	5' integration forward
	7726	AACCTCGGGGCATTCCTTG	3' integration reverse
PPCDC-Ty	6253	GGCGGTACCGCCTCTGCGACAATTTAGTG	FW cloning 3' *PPCDC*
	6254	GGCATGCATAGAACCTTTGTGTCTGCATAG	RV cloning 3' *PPCDC*
DPCK-iKD	7960	GTCTTCCGACAGTTTCCTAAAGTTTTAGAGCTAGAAATAGC	gRNA cloning
	8247	GCAAGAGTTCATTGCTGCCCAAAAAGCCGACATGTTTGCGGATCCGGGG	PCR homology template
	8256	CCAAAGGAATAACACTACGGCGGTGATCGCCATTTTGATATCCCTAGGAATTCACTCG	PCR homology template
	8249	GGACAACGAACGGGGCAAG	5' integration forward
	8250	AGCCATCCAAAGCAGAAGCC	3' integration reverse

DPCK-SMmyc	8251	GCTACGGTTGGTGCCGTTGTGTTTTAGAGCTAGAAATAGC	gRNA cloning
	9153	CAGGGCATTGCGTACTTTAGGGGGCGTATAATGGAGCAAAAACTGATATCG	PCR homology template
	9154	CCCGCCGATTGTCACCGAGAGGATTAACCAGGGCGAATTGGAGCTCC	PCR homology template
cDPCK	9046	CTTGAATTCCAGAAAAATGGCGATCACCGCCG	Fw cDNA cloning
	9047	TTCGATATCTATACGCCCCCTAAAGTACGC	Rv cDNA cloning
PBAL-Ty	7793	AATGGGCCCCCGCCTCTCTTCAACACGC	Fw cloning 3' *PBAL*
	7794	AACCCTGCAGGCAGCCTCAGGTCCGTCAC	Rv cloning 3' *PBAL*
PBAL-ko	7228	GTAAATGGGGATGTCAAGTTGACCAACATTCGCTAAATCTGGTTTTAGAGCTAGAAATAGC	2gRNA cloning
	7229	GCTATTTCTAGCTCTAAAACAACGAAGCGCAGGGTTGCTAAACTTGACATCCCCATTTAC	2gRNA cloning
	7230	ATCGAGCGCGACTCCCCCGAGCTTGGGTGTTTGCATTCAAACCCGCCCGC	PCR homology template
	7231	ACTTTCTCGTACCGCACTGCAGAGCTGACTGGCGGCCGCTCTAGAAC	PCR homology template
	7232	CAGGTGCAAAGGCTAGGAC	5' integration forward
	7233	TCTCTACACACCGGAGCTTC	3' integration reverse
	7628	CGAGCTCCACAGAAAACTGC	5' integration reverse
KPHMT-KPR-SM-Myc	9200	GTGATTGTCGCCACTCAAACGTTTTAGAGCTAGAAATAGC	gRNA cloning
	9381	ATGAATCTCGTCAAGTTCATCACCGAGCGAATGGAGCAAAAACTGATATCG	KOD homology template
	9382	TGGGCAGGATGAAGAGCTACGAGGACATGCGGGCGAATTGGAGCTCC	KOD homology template
KPHMT-KPR-ko	9374	GTAAATGGGGATGTCAAGTTGTGTTGGCTGCTGGGGCACAGGTTTTAGAGCTAGAAATAGC	2gRNA cloning
	9375	GCTATTTCTAGCTCTAAAACGTTTGAGTGGCGACAATCACAACTTGACATCCCCATTTAC	2gRNA cloning
	9376	TTTGAATGACACAGTAGTCCTTGCTCTGTAGCGGAAGATCCGATCTTGC	KOD homology template
	9377	GCACACTGACACTGCGCCATTTACGCGGTCGCGGCCGCTCTAGAACTAG	KOD homology template
	9378	CTATGCCTTGCTGTCAGCG	5' integration forward
	9379	TGGGGAACACACAGTAGCG	3' integration reverse
	9380	AAGCGTGGCCAACAAGG	5' integration reverse
c*Tg*PBAL-myc	10121	CTTGAATTCCCTTTTTCGACAAAATGGACACCTCAGATTTAGCG	cDNA cloning *TgPBAL*
	9156	TTCGATATCCAGCCTCAGGTCCGTCAC	cDNA cloning *TgPBAL*
c*Ec*PBAL-myc	10126	CTTGAATTCCCTTTTTCGACAAAATGTTAATTATCGAAACCCTGCCGC	cDNA cloning *EcPBAL*
	10127	TTCGATATCCGCCAGCTCGACCATTTTG	cDNA cloning *EcPBAL*
	8414	CTTCAGCACACCACCCGTTCAG	3' integration uprt
	9318	GAAGAACGACGCTGCAAAC	uprt locus fw
	9319	TTGCCATGTCAAGTTCCTACC	uprt locus rv

PBAL-cd	10166	CCACCAATGCATGCGCAGCGACCTCGCCG	clone full catalytic domain *PBAL* in pStrep
	10167	CGTGGTACCTTAGGACCCTGTCCCTGGC	clone full catalytic domain *PBAL* in pStrep
	9975	TGTACTTCCAATCCAATGCATGCGACACCTCAGATTTAGCGAATGTTG	clone full length *PBAL* in pStrep
	9976	ACGCGTGGTACCGGCCGGCCTTAAAGCCTCAGGTCCGTCAC	clone full length *PBAL* in pStrep
universal primers	5370	GCCGTAGTCTTCAATGGGTTTGG	HXGPRT reverse
	1629	CAGTGACACCGCGGTGGAGG	HXGPRT forward
	7081	GGGTCGAGCCCGAGCCCT	mAID reverse
	4609	CGACAACACCTTCTACAACGCTG	HXGPRT fw
	3596	GGCCTTCGATACCGACTTCATTGAG	tet reverse
	1935	cgctgcaccacttcattatttcttctgg	sag1 3' forward
	2074	CCGTAGTCTTCAATGGGTTTGGACGC	HXGPRT reverse
	2018	CTTGGGGGTCATCGCGACGACCAGAC	DHFR rv
	2017	GTCACTTGTTGTGCCAGTTCTAC	DHFR fw
	9318	GAAGAACGACGCTGCAAAC	uprt locus
	9319	TTGCCATGTCAAGTTCCTACC	uprt locus
	3979	ACACAAGGTGATTGTGTAACACCG	tubulin promoter rv

Supplementary Methods

liquid chromatography conditions and mass spectrometry parameters

Reversed-phase UPLC-MS conditions

Quantification of Pan, PPan-Cys, deP-CoA and CoA (method A) was performed on the Q Exactive Plus analytical platform as follows: 10 µl of sample (6°C) were injected and eluted with a tailored gradient: 0%B (1 min), 5-25%B in 7 min, 25-40%B in 1 min and hold 3 min, wash at 95%B (total runtime of 20 min). Here and in all other methods, blanks (ultrapure water or 80% acetonitrile for HILIC analyses) were injected after every sample to avoid carry-over. Eluent A was 50 mM ammonium acetate buffer (pH 6.9) and eluent B was acetonitrile. Flow rate and column oven temperature were respectively of 250 µl/min and 30 °C. The mass spectrometer was operated in positive polarity with a heated electrospray ionization (HESI-II) probe. Electrospray voltage was set to 3500 V; the sheath gas, auxiliary gas and sweep gas were set, respectively, to 46, 11 and 2 (arbitrary units, nitrogen). The auxiliary gas heater and transfer capillary temperatures were of 350 °C and 300 °C. PRM experiments were looped for each precursor with an isolation window of 0.7u, a normalized collision energy of 30 eV. All experiments were acquired in profile mode at 17.5 k resolution with an AGC of 1e6 and a fill time of 50 ms (1 µscan).

For labelling experiments (method B - Fig. 5f), 5 µl of sample (6°C) were injected and separated with a linear gradient from 0%B (1 min hold) to 95%B in 14 min (total runtime of 20 min after column wash and reconditioning). Eluent A was 5 mM ammonium formate with 0.25% formic acid (pH 2.8) and eluent B was acetonitrile. Flow rate and column oven temperature were respectively of 250 µl/min and 35 °C. Mass spectrometry experiments were a full scan MS looped with PRM (FS-PRM) experiments for each precursor (isol. window of 0.7u - NCE = 25). Electrospray interface settings were as described above, and MS data was acquired in profile mode at 35 k resolution with an AGC of 1e6 and a fill time of 100 ms (1 µscan).

For the QTRAP 3200 analytical platform, 10 µl of sample (8°C) were injected and eluted with the same gradient as for method A with the Q Exactive Plus instrumentation. The mass spectrometer was operated in positive polarity with a TurboV ion source fitted with a TurboIonSpray probe (SCIEX). The electrospray voltage was set to 5500 V, the nebulizer gas and auxiliary gas (nitrogen) were set to 40 and 50 psi, respectively. The auxiliary gas heater was set to 500 °C and the curtain plate (CUR), entrance lens (EP) and collision cell exit (CXP) potentials were of 10 V. The declustering potential (DP) was set to 70 V. The MRM transitions of the precursor ion (Q1) and product ion (Q3), as well as the collision energy (CE) are summarized in the table (Q1 and Q3 were operated at unit resolution):

**Name**	**Q1 (m/z)**	**Q3 (m/z)**	**Dwell (ms)**	**CE (eV)**
β-Ala_1	90.1	44.0	30	40
$^{13}C_3/^{15}N$-β-Ala_1	94.1	47.0	30	40
β-Ala_2	90.1	55.0	30	30
$^{13}C_3/^{15}N$-β-Ala_2	94.1	58.0	30	30
Panthotenate_1	220.1	90.0	30	35
$^{13}C_3/^{15}N$-panthotenate_1	224.1	94.2	30	35
Panthotenate_2	220.1	71.9	30	45
$^{13}C_3/^{15}N$-panthotenate_2	224.1	75.9	30	45
CoA_1	768.1	261.0	30	26
$^{13}C_3/^{15}N$-CoA_1	772.1	265.0	30	26
CoA_2	768.1	428.0	30	23
$^{13}C_3/^{15}N$-CoA_2	772.1	428.0	30	23
Acetyl-CoA_1	810.1	303.0	30	27
$^{13}C_3/^{15}N$-acetyl-CoA_1	814.1	307.0	30	27

Acetyl-CoA_2	810.1	428.0	30	24
$^{13}C_3/^{15}N$-acetyl-CoA_2	814.1	428.0	30	24

For the analyses performed with the QTRAP 6500, the following UPLC-MS conditions were used: 8 µl of sample (6°C) were injected and eluted with the same gradient and column as described for method A. The mass spectrometer was operated with the same settings as previously mentioned except the curtain plate (CUR) and collision cell exit (CXP) potentials set to 30 V and 8 V respectively. The declustering potential (DP) was of 90 V. The set of MRM transitions was the same with the addition of two transitions to monitor dephospho-CoA:

**Name**	**Q1 (m/z)**	**Q3 (m/z)**	**Dwell (ms)**	**CE (eV)**
Dephospho-CoA_1	688.2	261.1	30	26
Dephospho-CoA_2	688.2	348.1	30	23

HILIC UPLC-MS conditions

HILIC analyses were carried out on the Q Exactive Plus analytical platform as follows: 5 µl of sample (6°C) were injected and eluted with a gradient adapted [71]: 100%B held for 6 min and stepped down to 94.1%B, 94.1-82.4%B in 4 min, 82.4-70.6%B in 2 min and back to 100%B (total runtime of 20 min). Eluent A was 10 mM ammonium formate with 0.15% formic acid in water and eluent B was 10 mM ammonium formate with 0.15% formic acid in acetonitrile-water (85:15, v/v). Flow rate and column oven temperature were respectively of 400 µl/min and 40 °C. The mass spectrometer was operated in positive or negative polarity with a heated electrospray ionization (HESI-II) probe. Electrospray settings were as aforementioned. A full MS experiment was followed with PRM experiments looped for each precursor with an isolation window of 0.7u, a normalized collision energy of 30 eV. All experiments were acquired in profile mode at 17.5 k resolution with an AGC of 1e6 and a fill time of 50 ms (1 µscan).

Supplementary Figures raw blots and gels

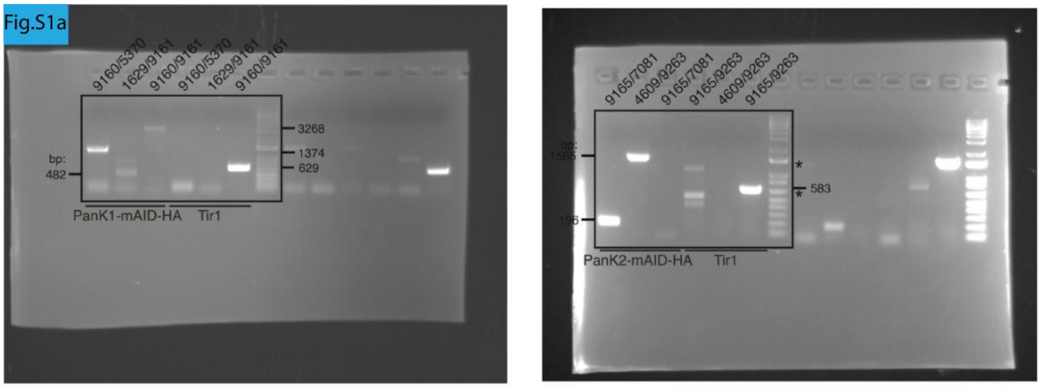

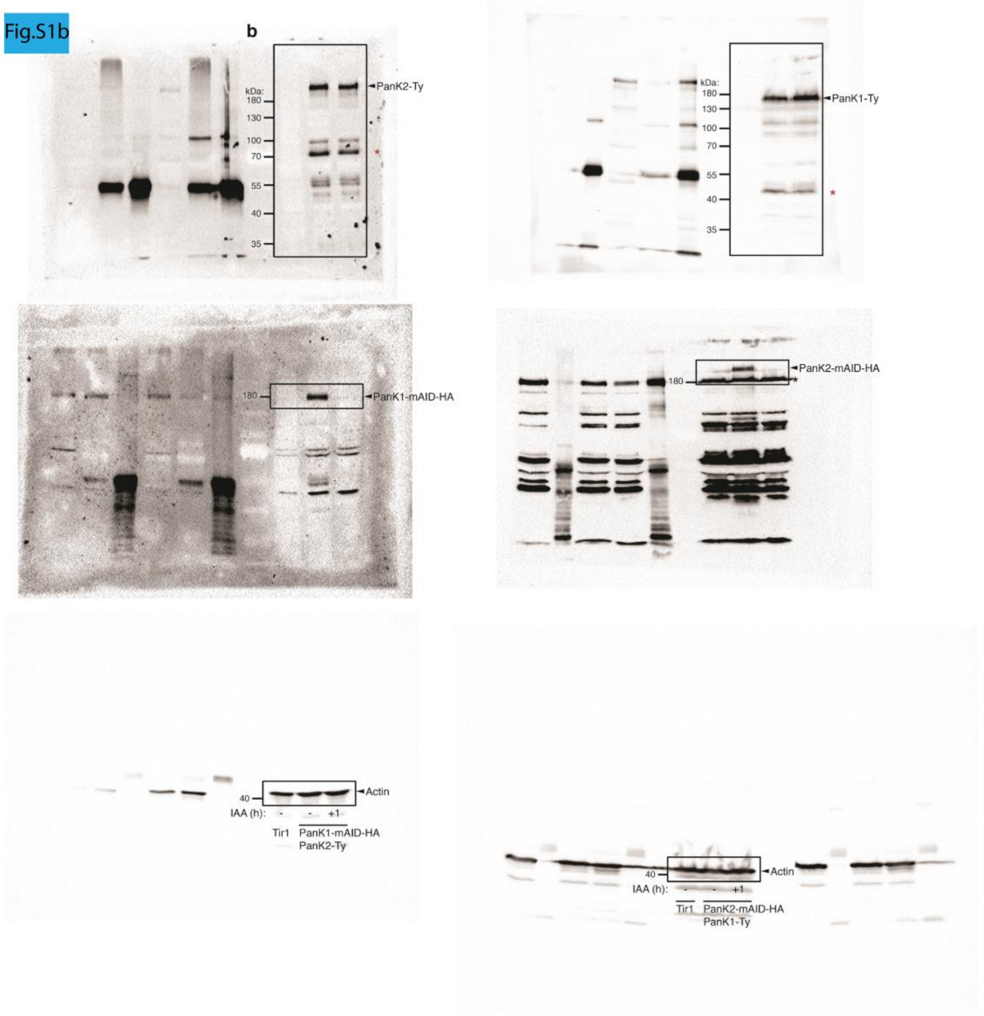

Fig.S1c

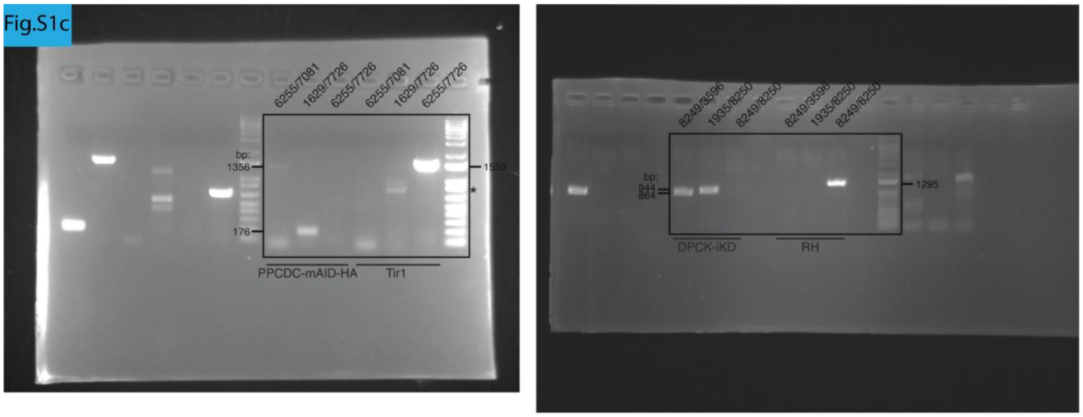

Fig.S2a

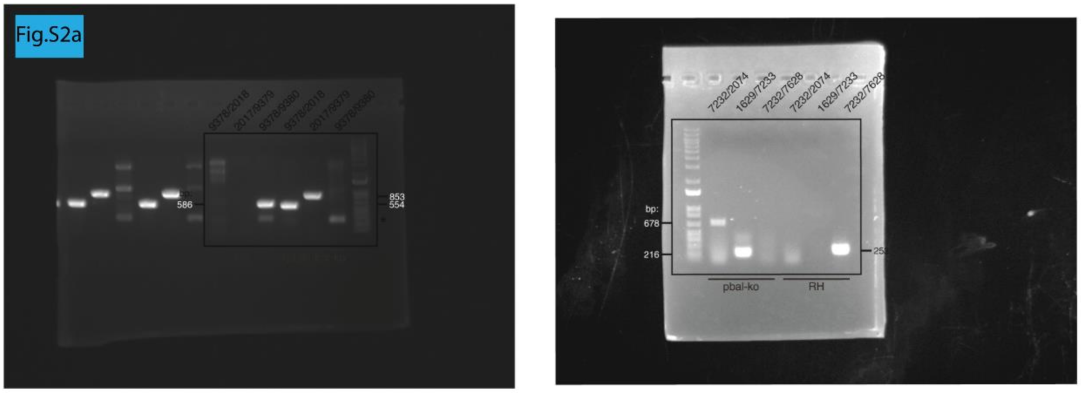

Fig.S6a

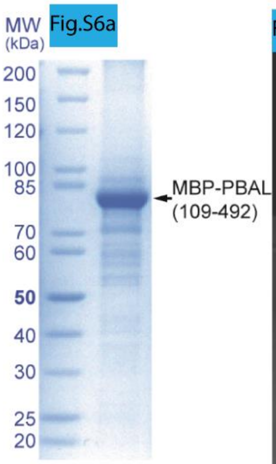

Fig.S9a

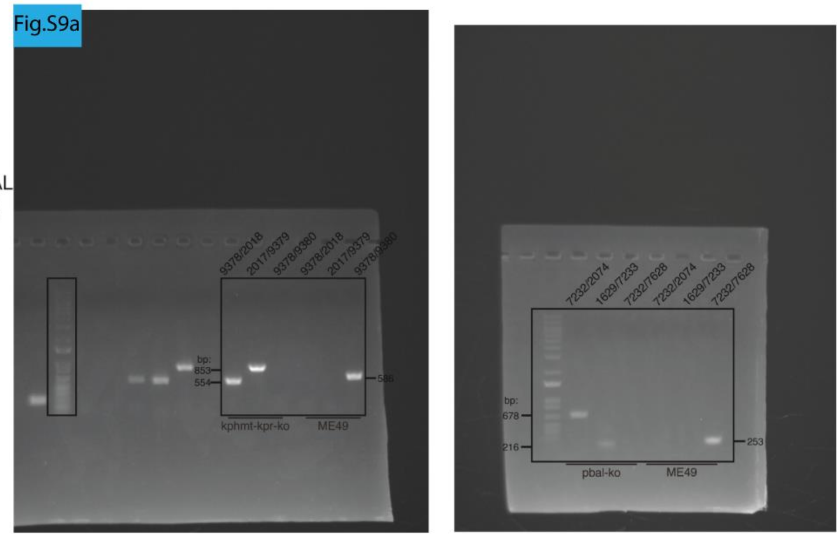

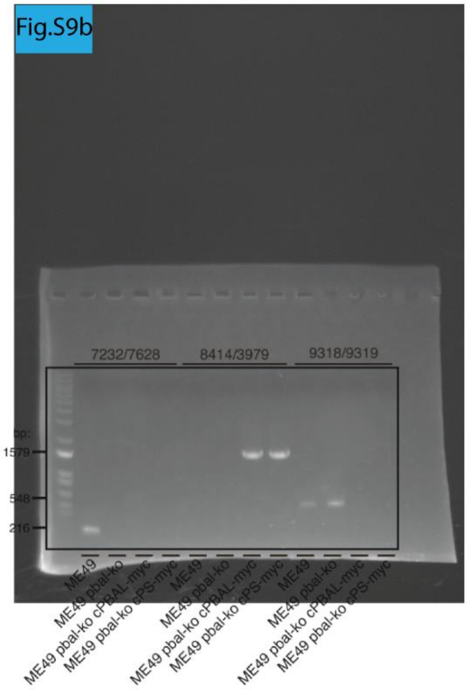
Fig.S9b
7232/7628
8414/3979
9318/9319
bp:
1579
548
216
ME49
ME49 pbal-ko
ME49 pbal-ko cPBAL-myc
ME49 pbal-ko cPS-myc
ME49
ME49 pbal-ko
ME49 pbal-ko cPBAL-myc
ME49 pbal-ko cPS-myc
ME49
ME49 pbal-ko
ME49 pbal-ko cPBAL-myc
ME49 pbal-ko cPS-myc

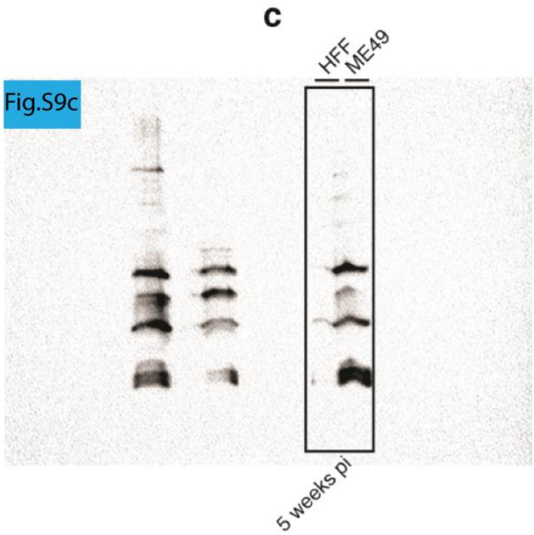
c
HFF
ME49
Fig.S9c
5 weeks pi
