# Supplementary material for: Pantothenate biosynthesis is critical for chronic infection by the neurotropic parasite Toxoplasma gondii
Source: Nat Commun. 2022 Jan 17;13:345. doi: 10.1038/s41467-022-27996-4 (PMC8764084; doi:10.1038/s41467-022-27996-4)
Supplement: Supplementary file 5 — Supplementary Data 3 [file 41467_2022_27996_MOESM5_ESM.pdf]

Muscle alignment of TgPBAL orthologues
Black shading represents exact match
In red catalytic residues from crystallography DOI: 10.1110/ps.0241803
In green shade if demonstrated essential for protein activity DOI: 10.1021/bi049676n

```
                                    10         20         30         40         50         60
                             ....|....| ....|....| ....|....| ....|....| ....|....| ....|....|
ETH_00031750                 ---MNS---- ---------- ---------- ---------- ---------- ----------  3
Vbra_16581                   ---MGGVRTL YASGGGG--- ---------- -VKSLKMRDL LRKKKERRKI TVITAYDFIG  43
Cvel_3471                    MSYVGGPTSS TSEGGGASSL CFGNFMSFEP QLKSLKLEDL LGRKKAGCKI TMITAYDYLS  60
SN3_00101300                 PETEETSS-- ---------- ---------- ---------- ---------- ------HQVP  12
NCLIV_039640                 ---METHT-- ---------- ---------- ---------- ---------- ----------  5
TGME49_265870                ---MDTSD-- ---------- ---------- ---------- ---------- ----------  5
HHA_265870                   ---MDTSD-- ---------- ---------- ---------- ---------- ----------  5
XP_002768075.P. marinus      ---------- ---------- ---------- ---------- ---------- ----------  1
XP_002767048.P. marinus      ---------- ---------- ---------- ---------- ---------- ----------  1
sp|P31663|PANC_ECOLI         ---------- ---------- ---------- ---------- ---------- ----------  1
sp|P9WIL5|PANC_MYCTU         ---------- ---------- ---------- ---------- ---------- ----------  1

                                    70         80         90        100        110        120
                             ....|....| ....|....| ....|....| ....|....| ....|....| ....|....|
ETH_00031750                 ---------- ---------- ---------- ---------- ---------P RRSEMLVLRT  14
Vbra_16581                   AQVANSAMLD CILVGDSLAN VVLGHARTNQ IGMDEMLYHC KSVTRGNSRC FVIGDMPFGS  103
Cvel_3471                    GRIADAAAID MVLVGDSLGN VVLGHSRTDK VTMEDMLHHT RAVTRGCKRA FVVGDLPFGS  120
SN3_00101300                 SQALRRTETS DLLLPLGDVV LRLGSPRRVT RSSASHIHEQ AYLRRF---A SAVLPFPAVS  69
NCLIV_039640                 ---------- ---------- --LAQLGRDT LP-------- ---------A FSAEFLPSSS  26
TGME49_265870                ---------- ---------- --LANVG--- -P-------- ---------Q FSVELGTRFA  22
HHA_265870                   ---------- ---------- --LANVG--- -S-------- ---------Q FSVELGTRFA  22
XP_002768075.P. marinus      ---------- ---------- ---------- ---------- ---------M LSLVPKPRSD  11
XP_002767048.P. marinus      ---------- ---------- ---------- ---------- ---------M LSLVPKPRSD  11
sp|P31663|PANC_ECOLI         ---------- ---------- ---------- ---------- ---------M LIIETLPL--  9
sp|P9WIL5|PANC_MYCTU         ---------- ---------- ---------- ---------- ---------M TIPAFHP---  8

                                   130        140        150        160        170        180
                             ....|....| ....|....| ....|....| ....|....| ....|....| ....|....|
ETH_00031750                 CREVSVHRST CGRVRE---- ---------- ---------- ---------- ----------  30
Vbra_16581                   YSTVDRAVQN AIRFVNEGGV AAVKIEGPMY EQIKAVSQLT PVVGHTGVLP QTAENFLARG  163
Cvel_3471                    CCTVEKALEN AHALMRE-GC HAVKLEGPLL AQIQAISQFV PVVAHLGLQP QTASSFASRG  179
SN3_00101300                 SCFPCDAVIC NTSYST---- ---------- ------MYST SATAHHGTGP WAQENWDAFG  109
NCLIV_039640                 -AFF---PF- --RATA---- ---------- ---------- ---------- ----------  35
TGME49_265870                -AKFCVQRL- --NWSA---- ---------- ---------- ---------- ----------  34
HHA_265870                   -AKFCVQKL- --NLSA---- ---------- ---------- ---------- ----------  34
XP_002768075.P. marinus      ISELAAKISA RIAAKA---- ---------- ---------- ---------- ----------  27
XP_002767048.P. marinus      ISELAAKISA RITAKS---- ---------- ---------- ---------- ----------  27
sp|P31663|PANC_ECOLI         ---------- ---------- ---------- ---------- ---------- ----------  9
sp|P9WIL5|PANC_MYCTU         ---------- ---------- ---------- ---------- ---------- ----------  8

                                   190        200        210        220        230        240
                             ....|....| ....|....| ....|....| ....|....| ....|....| ....|....|
ETH_00031750                 ---------- ---------- ---------- ---------- ---------- ----------  30
Vbra_16581                   KTAKDAQYLV EEAQALEAAG CCMIVLEKVC SEVAEEISRR LTIPTIGIGA GPHCDGQVLV  223
Cvel_3471                    RTADEAITLL EDARAVEAAG ASFLVLEKVA AEVAQEITSS LSIPTIGIGA GNVTDGQVLV  239
SN3_00101300                 SGCLPWSRSN FPFSADCATE DAPLSTSSYM SAPSPPESGV LDACHTKAAE RPRNVTGLQK  169
NCLIV_039640                 ---------- ---------- -----PPPRD R--------- ---------- ----------  41
TGME49_265870                ---------- ---------- -----NRKIC R--------- ---------- ----------  40
HHA_265870                   ---------- ---------- -----NQEIC R--------- ---------- ----------  40
XP_002768075.P. marinus      ---------- ---------- ---------- ---------- ---------- ----------  27
XP_002767048.P. marinus      ---------- ---------- ---------- ---------- ---------- ----------  27
sp|P31663|PANC_ECOLI         ---------- ---------- ---------- ---------- ---------- ----------  9
sp|P9WIL5|PANC_MYCTU         ---------- ---------- ---------- ---------- ---------- ----------  8

                                   250        260        270        280        290        300
                             ....|....| ....|....| ....|....| ....|....| ....|....| ....|....|
ETH_00031750                 ---------- ---------- -------GFP ASCPILAATP EQQQQQ---- ----------  49
Vbra_16581                   WHDVLGLQSP GSLSLKFVRR FGD----GWR QTAKAVEEYA TAVERETFPH PQHNSFLMDP  279
Cvel_3471                    WHDVLGLCPP GFPKIKFAKT FGGEPGWGWK AALHAVEEYA EAVTRGIFPG -QPQTFYMGA  298
SN3_00101300                 QHPCAPSQPP RTP------- -------RPI VLIHSPRELL -AYRNA---- ----------  200
NCLIV_039640                 ---------- ---------- -------REI LVVHSPQELV -AYRNA---- ----------  59
TGME49_265870                ---------- ---------- -------SSH VRVLNPRNCP RNCRNG---- ----------  59
HHA_265870                   ---------- ---------- -------SSQ VRVLNPRNFP RSHRNG---- ----------  59
XP_002768075.P. marinus      ---------- ---------- -------GPP VVVRSVGDFV ALHNTD---- ----------  46
XP_002767048.P. marinus      ---------- ---------- -------GPP VVVRSVGDFV ALHNTD---- ----------  46
sp|P31663|PANC_ECOLI         ---------- ---------- ---------- --------LR QQIRRL---- ----------  17
sp|P9WIL5|PANC_MYCTU         ---------- ---------- -------GEL NVYSAPGDVA DVSRAL---- ----------  27

                                   310        320        330        340        350        360
```

```
                              ....|....| ....|....| ....|....| ....|....| ....|....| ....|....|
ETH_00031750                  ---------- ---------- ---------- ---------- ---------- ----------  49
Vbra_16581                    GERDAFIRWT EGEPLSPA-- ---------- ----DVVVDQ G--------- ----------  304
Cvel_3471                     AAREDFEKRR GGRLLPPACN LRGAVGGSAS SPGVTPVSGQ GDGSTPPCSE PWRPPSTATG  358
SN3_00101300                  ---------- ---------- ---------- ---------- ---------- ----------  200
NCLIV_039640                  ---------- ---------- ---------- ---------- ---------- ----------  59
TGME49_265870                 ---------- ---------- ---------- ---------- ---------- ----------  59
HHA_265870                    ---------- ---------- ---------- ---------- ---------- ----------  59
XP_002768075.P. marinus       ---------- ---------- ---------- ---------- ---------- ----------  46
XP_002767048.P. marinus       ---------- ---------- ---------- ---------- ---------- ----------  46
sp|P31663|PANC_ECOLI          ---------- ---------- ---------- ---------- ---------- ----------  17
sp|P9WIL5|PANC_MYCTU          ---------- ---------- ---------- ---------- ---------- ----------  27

                                     370        380        390        400        410        420
                              ....|....| ....|....| ....|....| ....|....| ....|....| ....|....|
ETH_00031750                  ---------- ---------- ---------- ---------- ---------- ----------  49
Vbra_16581                    ---------- ---------- ---------- ---------- ---------- ----GEPALS  310
Cvel_3471                     RHAQAQGESA PFESVGSPSR IHGGVGLANG GGTGGVRANG GNEMLRNVAN GGSAGPPVIV  418
SN3_00101300                  ---------- ---------- ---------- ---------- ---------- ----RPPVYV  206
NCLIV_039640                  ---------- ---------- ---------- ---------- ---------- ----RPRV--  63
TGME49_265870                 ---------- ---------- ---------- ---------- ---------- ----NTRQS-  64
HHA_265870                    ---------- ---------- ---------- ---------- ---------- ----NTRQS-  64
XP_002768075.P. marinus       ---------- ---------- ---------- ---------- ---------- ----------  46
XP_002767048.P. marinus       ---------- ---------- ---------- ---------- ---------- ----------  46
sp|P31663|PANC_ECOLI          ---------- ---------- ---------- ---------- ---------- ----------  17
sp|P9WIL5|PANC_MYCTU          ---------- ---------- ---------- ---------- ---------- ----------  27

                                     430        440        450        460        470        480
                              ....|....| ....|....| ....|....| ....|....| ....|....| ....|....|
ETH_00031750                  ---------- ---------- ---------- ---------- -LLLLQQQQQ QGRCEGC---  65
Vbra_16581                    ASGVGGMVAG GLKRVLVIGS GGMASLVAYM LATKGDVAVT VATRWQQQAS AISSGGGLSC  370
Cvel_3471                     PNGPGGGMSL GFRRVAVVGC GSLGKLVGWS IAGVTGVKVS VVCRRQEQVD RILTRGGIEC  478
SN3_00101300                  HHHCCGDNLH SHHNSFPSSF TRDSCDNCSD FHFDHSACPG CSPSSSKFVP PFASVGGQKY  266
NCLIV_039640                  ---------- ----QLPV-- ---------- ----VAVRAA ---------- -GRTEGGAAA  82
TGME49_265870                 ---------E ERQRRLPT-- ---------- ----RTAGAA LPVRARSLVN QEPNGGGPAS  99
HHA_265870                    ---------E ERQRRLPT-- ---------- ----RTVGAA LPVRARSLVI QEPNGGGPAS  99
XP_002768075.P. marinus       ---------- ---------- ---------- ---------- ---------- ----------  46
XP_002767048.P. marinus       ---------- ---------- ---------- ---------- ---------- ----------  46
sp|P31663|PANC_ECOLI          ---------- ---------- ---------- ---------- ---------- ----------  17
sp|P9WIL5|PANC_MYCTU          ---------- ---------- ---------- ---------- ---------- ----------  27

                                     490        500        510        520        530        540
                              ....|....| ....|....| ....|....| ....|....| ....|....| ....|....|
ETH_00031750                  ---------- ---------- ---------- ---------- ---------- ----------  65
Vbra_16581                    VDPLGNPHG- PPVTLDVVTD DTTLPQGAFD LAIILIKSAD TERAAKLARR AVRPDGCVLS  429
Cvel_3471                     EDADGKSAGV REVTAGVASD ---FHSGSAD LVVVAVKSFD TQGAAEIAAR LCAPEGAVLS  535
SN3_00101300                  VTVTDCSAS- ---------- ----TVGSVF QGDFTVGTHN VKAYQGASLE GDSCNSVLSA  311
NCLIV_039640                  ADSTGASAS- ---------- ----ESGTG- ---------- ---------A GDSVDALTHA  107
TGME49_265870                 ADAT----S- ---------- ----SPGRS- ---------- ---------A ATSPNASLLS  120
HHA_265870                    ADAT----S- ---------- ----SPGRS- ---------- ---------A ATWPNASLLS  120
XP_002768075.P. marinus       ---------- ---------- ---------- ---------- ---------- ----------  46
XP_002767048.P. marinus       ---------- ---------- ---------- ---------- ---------- ----------  46
sp|P31663|PANC_ECOLI          ---------- ---------- ---------- ---------- ---------- ----------  17
sp|P9WIL5|PANC_MYCTU          ---------- ---------- ---------- ---------- ---------- ----------  27

                                     550        560        570        580        590        600
                              ....|....| ....|....| ....|....| ....|....| ....|....| ....|....|
ETH_00031750                  ---------- ---------- ---------- ---------- ---------- -------VAP  68
Vbra_16581                    LQNGLDAPLE LKKAF----K GRDGPKLLLG TTTNGALVRA G--------- DEGRVWRMGE  476
Cvel_3471                     LQNGLSHVGV LRKALAKSAR SAGGPCLLLG TCTAGAAPGA ADGDGSGGAV DPSRVSFRGR  595
SN3_00101300                  PENEYPIEQA TEKKVSSLNS HPIRDGCPAL RVQNTSKGGL RDTCLSTRQR GYDSQSHYIR  371
NCLIV_039640                  HV-------- ---------- ---------- ---------- ---------- --DRSSMFSR  117
TGME49_265870                 PS-------- ---------- ---------- ---------- ---------- --SPLAFSSR  130
HHA_265870                    PS-------- ---------- ---------- ---------- ---------- --SPLASSSR  130
XP_002768075.P. marinus       ---------- ---------- ---------- ---------- ---------- -------VFR  49
XP_002767048.P. marinus       ---------- ---------- ---------- ---------- ---------- -------VFR  49
sp|P31663|PANC_ECOLI          ---------- ---------- ---------- ---------- ---------- -------RME  20
sp|P9WIL5|PANC_MYCTU          ---------- ---------- ---------- ---------- ---------- -------RLT  30

                                     610        620        630        640        650        660
                              ....|....| ....|....| ....|....| ....|....| ....|....| ....|....|
ETH_00031750                  RR-------- ---------- ---------- ---------- ---------- ----------  70
Vbra_16581                    GETVIG---- --------AA ERKDEACVER VRDLLNAAAL TTRTVRSQDV LETIWHKLAI  524
Cvel_3471                     GRFVVGLMGD GEGFQRGRGG ASGPGEVAEA AASLLRSAEM DAEAVSEQEV LPALWLKVAV  655
SN3_00101300                  RR-------- ---------- ---------- ---------- ---------- ----------  373
NCLIV_039640                  RR-------- ---------- ---------- ---------- ---------- ----------  119
TGME49_265870                 RR-------- ---------- ---------- ---------- ---------- ----------  132
```

```
HHA_265870                  RR-------- ---------- ---------- ---------- ---------- ----------  132
XP_002768075.P. marinus     GL-------- ---------- ---------- ---------- ---------- ----------  51
XP_002767048.P. marinus     GL-------- ---------- ---------- ---------- ---------- ----------  51
sp|P31663|PANC_ECOLI        GK-------- ---------- ---------- ---------- ---------- ----------  22
sp|P9WIL5|PANC_MYCTU        GR-------- ---------- ---------- ---------- ---------- ----------  32

                                   670        680        690        700        710        720
                            ....|....| ....|....| ....|....| ....|....| ....|....| ....|....|
ETH_00031750                ---------- ---------- ---------- ---------- ---------- ----------  70
Vbra_16581                  NAVVNPLTAL YGVNNGALTR PHFEPLISQI AQEVSEVGRA RGIRLPEGSN LVKVVQRAAE  584
Cvel_3471                   NAAINPLTAL MGICNGGLET QPLSTVAAAI AEEVRVVARS KGVRLSEAS- VRSALESTIR  714
SN3_00101300                ---------- ---------- ---------- ---------- ---------- ----------  373
NCLIV_039640                ---------- ---------- ---------- ---------- ---------- ----------  119
TGME49_265870               ---------- ---------- ---------- ---------- ---------- ----------  132
HHA_265870                  ---------- ---------- ---------- ---------- ---------- ----------  132
XP_002768075.P. marinus     ---------- ---------- ---------- ---------- ---------- ----------  51
XP_002767048.P. marinus     ---------- ---------- ---------- ---------- ---------- ----------  51
sp|P31663|PANC_ECOLI        ---------- ---------- ---------- ---------- ---------- ----------  22
sp|P9WIL5|PANC_MYCTU        ---------- ---------- ---------- ---------- ---------- ----------  32

                                   730        740        750        760        770        780
                            ....|....| ....|....| ....|....| ....|....| ....|....| ....|....|
ETH_00031750                ---------- ---------- ---------- ---------- ---------- ----------  70
Vbra_16581                  TTAANTSSMR TDVENRLPTE ISAICGAIAS EARKVGLSAP VNEMLVHLIK A---LEEQRQ  641
Cvel_3471                   GTARNQSSML QDVQRGSQSE VRAICGAVAE EARRNGVAAP LCSLLCLMVE GKLKLQQQPQ  774
SN3_00101300                ---------- ---------- ---------- ---------- ---------- ----------  373
NCLIV_039640                ---------- ---------- ---------- ---------- ---------- ----------  119
TGME49_265870               ---------- ---------- ---------- ---------- ---------- ----------  132
HHA_265870                  ---------- ---------- ---------- ---------- ---------- ----------  132
XP_002768075.P. marinus     ---------- ---------- ---------- ---------- ---------- ----------  51
XP_002767048.P. marinus     ---------- ---------- ---------- ---------- ---------- ----------  51
sp|P31663|PANC_ECOLI        ---------- ---------- ---------- ---------- ---------- ----------  22
sp|P9WIL5|PANC_MYCTU        ---------- ---------- ---------- ---------- ---------- ----------  32

                                   790        800        810        820        830        840
                            ....|....| ....|....| ....|....| ....|....| ....|....| ....|....|
ETH_00031750                ---------- ---------- ---------- ---------- ---------- ----------  70
Vbra_16581                  T--------- -----TPPAP ASPPLPPQPT AAKETHAALA PTAAATSPPM ATRVCKTPSE  687
Cvel_3471                   RQQQEQHSKS STYDGSSPAC VSHPSPSSNG LQANGHAEYV SAGAKVSHTA RTTLVRDPKK  834
SN3_00101300                ---------- ---------- ---------- ---------- ---------- ----------  373
NCLIV_039640                ---------- ---------- ---------- ---------- ---------- ----------  119
TGME49_265870               ---------- ---------- ---------- ---------- ---------- ----------  132
HHA_265870                  ---------- ---------- ---------- ---------- ---------- ----------  132
XP_002768075.P. marinus     ---------- ---------- ---------- ---------- ---------- ----------  51
XP_002767048.P. marinus     ---------- ---------- ---------- ---------- ---------- ----------  51
sp|P31663|PANC_ECOLI        ---------- ---------- ---------- ---------- ---------- ----------  22
sp|P9WIL5|PANC_MYCTU        ---------- ---------- ---------- ---------- ---------- ----------  32

                                   850        860        870        880        890        900
                            ....|....| ....|....| ....|....| ....|....| ....|....| ....|....|
ETH_00031750                ---------- --RIGFVPTM GGLHWGHLSL IRAAERH--- CDEVWVSIFL NPTQFQSQED  115
Vbra_16581                  LMGVRSTLPA SARVAFVPFL GGLHDGHMAL LDEASRR--- GDRVWTSLFL NQLQFQSAKD  744
Cvel_3471                   LTALRKQVPP SSRVVFVPFL GGLHHGHLAL VEDARRRCGE GGQVWCSVFL NRLQFQSAQD  894
SN3_00101300                ---------- --RISLVPTL GGLHEGHLHL MRQAACL--- SDEVWVSIFL NPLQFQSSKD  418
NCLIV_039640                ---------- --RISLVPTM GGLHEGHLHL IRGAACT--- GDEVWVTIFV NALQFHSAKD  164
TGME49_265870               ---------- --RISLVPTM GGLHEGHLHL IRGAACT--- GDEVWVTIFV NALQFHSAKD  177
HHA_265870                  ---------- --RISLVPTM GGLHEGHLHL IRGAACT--- GDEVWVTIFV NALQFHSAKD  177
XP_002768075.P. marinus     ---------- --SVGFVPTM GSLHSGHMKL IATARPN--- HDVLVVSIFV NPAQFSPEED  96
XP_002767048.P. marinus     ---------- --SVGFVPTM GSLHSGHMKL IATARPN--- HDVLVVSIFV NPAQFSPEED  96
sp|P31663|PANC_ECOLI        ---------- --RVALVPTM GNLHDGHMKL VDEAKAR--- ADVVVVSIFV NPMQFDRPED  67
sp|P9WIL5|PANC_MYCTU        ---------- --RVMLVPTM GALHEGHLAL VRAAKRV--P GSVVVVSIFV NPMQFGAGED  78

                                   910        920        930        940        950        960
                            ....|....| ....|....| ....|....| ....|....| ....|....| ....|....|
ETH_00031750                FDTYPIKVEE DLHLLTTQ-- TNAQVLFLPE ASEMYPHLAP RRGAPAGAPG GGPPVGAP--  171
Vbra_16581                  FETYPMRLDE DIAKLRDR-- N-VEVIFTPN VQDIYP--TA EDSF------ ----------  783
Cvel_3471                   FDTYPMDLQR DVQMLEEA-- G-VDVVFAPS SEGVYS--DK DAVF------ ----------  933
SN3_00101300                LLTYPASIDE DIRLIQQL-- GVVSVLFVPH REALFP-LDA PAGF---SAS PGVTSASASS  472
NCLIV_039640                FLTYPASIDD DMKLLSQV-- R-VDLVFIPS HSSLYP-LDS RPHL---IAG SEELYTVP--  215
TGME49_265870               FLTYPASIDD DMKLLSQV-- R-VDLVFIPS HSSLYP-LDR RPHL---IAG SEVLSTIPAR  230
HHA_265870                  FLTYPASIDD DMKLLSQV-- R-VDLVFIPS HSSLYP-LDR RPHL---IAG SEVLSTIPAR  230
XP_002768075.P. marinus     YEQYPRDLEG DLKKLETESA G-VDVVFAPE PADMYP-KNP RAIV------ ----------  138
XP_002767048.P. marinus     YEQYPRDLEG DLKKLETESA G-VDVVFAPE PADMYP-KNP RAIV------ ----------  138
sp|P31663|PANC_ECOLI        LARYPRTLQE DCEKLNKR-- K-VDLVFAPS VKEIYP-NGT ETHT------ ----------  107
sp|P9WIL5|PANC_MYCTU        LDAYPRTPDD DLAQLRAE-- G-VEIAFTPT TAAMYP-DGL RTTV------ ----------  118

                                   970        980        990       1000       1010       1020
```

```
                             ....|....| ....|....| ....|....| ....|....| ....|....| ....|....|
ETH_00031750                 GEGLAPGGRP TAGGPPVGAP AQRRGVPFAE ---------- ---------- ----------  201
Vbra_16581                   ---------- ---------- ---------- ---------- ---------- ----------  783
Cvel_3471                    ---------- ---------- ---------- ---------- ---------- ----------  933
SN3_00101300                 EHGIRAGHLT ACGERKESTS EEGRETASQD SETLVSVPPA AAACHKTAAA VGDSAGTVTP  532
NCLIV_039640                 -RGRDNG--- --DARANGLP NATD----KE ADRVVRSPVS GFKLERLDTH AGERDA----  261
TGME49_265870                GRGLEDG--- --GGRVTSGQ SDDEADAMEE ADRVELSPVD --RLNRGDAR AREVDG----  279
HHA_265870                   GRGLEDG--- --GGRVTSGQ SDDEADAMEE ADRVEVSPVD --RLNRGDAR GREVDG----  279
XP_002768075.P. marinus      ---------- ---------- ---------- ---------- ---------- ----------  138
XP_002767048.P. marinus      ---------- ---------- ---------- ---------- ---------- ----------  138
sp|P31663|PANC_ECOLI         ---------- ---------- ---------- ---------- ---------- ----------  107
sp|P9WIL5|PANC_MYCTU         ---------- ---------- ---------- ---------- ---------- ----------  118

                                   1030       1040       1050       1060       1070       1080
                             ....|....| ....|....| ....|....| ....|....| ....|....| ....|....|
ETH_00031750                 ---------- ---------- ---------- ---------- ---------- ----------  201
Vbra_16581                   ---------- ---------- ---------- ---------- ---------- ----------  783
Cvel_3471                    ---------- ---------- ---------- ---------- ---------- ----------  933
SN3_00101300                 SVKLEGSSEL FLQGNDGDRL PSKVAATLGS VVYDKASPAR SVLTATSTPT AEESSAHEKG  592
NCLIV_039640                 ---------- ---------- ---------- ---LGPTPA- ----ASWIP- ----------  272
TGME49_265870                ---------- ---------- ---------- ---DGCSHS- ----A-WSP- ----------  289
HHA_265870                   ---------- ---------- ---------- ---DDCSHS- ----A-WSP- ----------  289
XP_002768075.P. marinus      ---------- ---------- ---------- ---------- ---------- ----------  138
XP_002767048.P. marinus      ---------- ---------- ---------- ---------- ---------- ----------  138
sp|P31663|PANC_ECOLI         ---------- ---------- ---------- ---------- ---------- ----------  107
sp|P9WIL5|PANC_MYCTU         ---------- ---------- ---------- ---------- ---------- ----------  118

                                   1090       1100       1110       1120       1130       1140
                             ....|....| ....|....| ....|....| ....|....| ....|....| ....|....|
ETH_00031750                 ---------- ---------- -LVVDFDGIE EVEGEGKRRP GFFR------ ----------  224
Vbra_16581                   ---------- ---------- PARVDFAGIE AVDGEGKERP GFFRGIGTVL TKFFAWTRPS  823
Cvel_3471                    ---------- ---------- PARIDFEGVE SVGGEGNMRP GFFKGVGTVV GKFFAWVRPD  973
SN3_00101300                 NSGRRRQQEK PNSSGSSRLF RMRVFFKDIE DVQGEGRRRR GFFGGIGTVV AQLLTLTRPT  652
NCLIV_039640                 ---------- -----PQRLF RMRVDFEGIE EVEGEGRRRS GFLRGIGTVV TQLFTLIRPT  317
TGME49_265870                ---------- -----PQRLF RMRVDFEGIE DVEGEGRRRC GFFRGIGTVV VQLFTLIRPT  334
HHA_265870                   ---------- -----PQRLF RMRVDFEGIE DVEGEGRRRC GFFRGIGTVV VQLFTLIRPT  334
XP_002768075.P. marinus      ---------- ---------- PSVTVEPNFV NGLSEAACRP TFFRGVATVV MKLFNIIRPE  178
XP_002767048.P. marinus      ---------- ---------- PSVTVEPNFV NGLSEAACRP TFFRGVATVV MKLFNIIRPE  178
sp|P31663|PANC_ECOLI         ---------- ---------- --YVDVPGLS TML-EGASRP GHFRGVSTIV SKLFNLVQPD  144
sp|P9WIL5|PANC_MYCTU         ---------- ---------- -----QPGPL AAELEGGPRP THFAGVLTVV LKLLQIVRPD  153

                                   1150       1160       1170       1180       1190       1200
                             ....|....| ....|....| ....|....| ....|....| ....|....| ....|....|
ETH_00031750                 ---------- --------VS SGCIDTRVVL CPTARG-PQG LPFASRNKRL TQQQLEKAQE  265
Vbra_16581                   VVVFGQKDFL QTVIVRKLCA EFFPDVEVAV VPTVRE-KDG LAFASRNARL TPEERKRAPL  882
Cvel_3471                    VAIFGQKDFL QTVVVKRLAR EFFIDTEIVV HETVRE-ESG LATASRNEKL TKEERHKAAG  1032
SN3_00101300                 YVHVGLKDFQ QVICLKRLVC GLGLDVLVVA HSTKRHASDG LSLSSRNKRL SGAERTAAAK  712
NCLIV_039640                 YAHFGFKDFQ QVACVKRLIC GMGLDALLLA HNTWRD-SDG MAASSRNRRM TEEDRKKARK  376
TGME49_265870                YAHCGFKDFQ QVACLKRLIC GMGLDALLLA HNTWRD-SDG VAASSRNRRM AEADRQKARK  393
HHA_265870                   YAHCGFKDFQ QVACLKRLIC GMGLDALLLA HNTWRD-SDG VAASSRNRRM AEADRQKARK  393
XP_002768075.P. marinus      RAYFGQKDAM QVSVIISMVK DLNVPVELEV VPTARE-ADG LASSSRNVYL TPAMREKATI  237
XP_002767048.P. marinus      RAYFGQKDAM QVSVIISMVK DLNVPVELEV VPTARE-ADG LASSSRNVYL TPAMREKATI  237
sp|P31663|PANC_ECOLI         IACFGEKDFQ QLALIRKMVA DMGFDIEIVG VPIMRA-KDG LALSSRNGYL TAEQRKIAPG  203
sp|P9WIL5|PANC_MYCTU         RVFFGEKDYQ QLVLIRQLVA DFNLDVAVVG VPTVRE-ADG LAMSSRNRYL DPAQRAAAVA  212

                                   1210       1220       1230       1240       1250       1260
                             ....|....| ....|....| ....|....| ....|....| ....|....| ....|....|
ETH_00031750                 ACAVLKEVEA LYNKGTR-QI GALRAAA--- AAAAERVG-A HLEYLAFNRQ ADGAPIVPLG  320
Vbra_16581                   IYQTLQAVEA QHRRGER-DA GVLTEAG--- RVFAASHG-L SVDYITVCDL YTGKAADVLQ  937
Cvel_3471                    LFASLSLVKD AVESGEVTSV SDLISLG--- LTALKKRE-M HMDYLILSDL ESGRELQEDE  1088
SN3_00101300                 YYSVLQQVCR SYERGER-RV QQLETEA--- RLHAAAAG-L HLYYITFNRW QDGAPVAYTS  767
NCLIV_039640                 SFLLLQHVSR -----ER--- ---------- ---------- ---------- ----------  388
TGME49_265870                SFLLLQHVGE VFRKGER-RV HRLLEEA--- RAFAEKEN-L LLHYTAIDRL EDGEPVVYRH  448
HHA_265870                   SFLLLQHVGE VFRKGER-RV HTLLEEA--- RAFAEKEN-L LLHYTAIDRL EDGEPVVYRH  448
XP_002768075.P. marinus      LYKSLCAAYD MVKSASK-PV KASEVEDVVK RTLLAEPMVL GIEYISVASV ETAQEVETIQ  296
XP_002767048.P. marinus      LYKSLCAAYD MVKSASK-PV KASEVEDVVK RTLLAEPMVL GIEYISVASV ETAQEVETIQ  296
sp|P31663|PANC_ECOLI         LYKVLSSIAD KLQAGER-DL DEIITIA--- GQELNEKG-F RADDIQIRDA DTLLEVSETS  258
sp|P9WIL5|PANC_MYCTU         LSAALTAAAH AATAGAQ--- -AALDAA--- RAVLDAAPGV AVDYLELRDI GLG-PMPLNG  264

                                   1270       1280       1290       1300       1310       1320
                             ....|....| ....|....| ....|....| ....|....| ....|....| ....|....|
ETH_00031750                 APRGAPRR-- ---------- ---------- ---------- ---------- -ASEGAPGGT  337
Vbra_16581                   PT-------- ---------- ---------- ---------- ---------- ----------  939
Cvel_3471                    EVCPGAAL-- ---------- ---------- ---------- ---------- ----------  1096
SN3_00101300                 GSSWTRKTPS CCSATSNTTD SVAPASCSSL GANQQEHTRQ QEQPAQQQLA RRKEDAEAQT  827
NCLIV_039640                 ---------- ---------- ---------- ---------- ---------- ----------  388
TGME49_265870                SGGAVKRIET QGESAEN--- ---------- --EERRAKRR RTESDIADET PLGRPGTGSP  493
```

```
HHA_265870               SGGAVKRIDT HRESAAN--- ---------- --GERRAKRR RTDSDIADEA QLGRPGTGSP  493
XP_002768075.P. marinus  FGPEA----- ---------- ---------- ---------- ---------- ----------  301
XP_002767048.P. marinus  FGPEA----- ---------- ---------- ---------- ---------- ----------  301
sp|P31663|PANC_ECOLI     ---------- ---------- ---------- ---------- ---------- ----------  258
sp|P9WIL5|PANC_MYCTU     SGR------- ---------- ---------- ---------- ---------- ----------  267

                                 1330       1340       1350       1360       1370       1380
                         ....|....| ....|....| ....|....| ....|....| ....|....| ....|....|
ETH_00031750             PNVDPTGGPT GAPRRAPCEA PEGPPQGDEG SLPEN-DQIC VAVAIR-MAP GCSIVDNFVL  395
Vbra_16581               ---------- ---------- ---------- ------KSYC VAIGAS-VGP SCRLVDNIVL  962
Cvel_3471                ---------- -CLHAGGSVK VSKP------ ---------V LLPSGT-PRE RVRLVDNVVI  1129
SN3_00101300             SRLQPEGEAN HERKVDGCEQ QQEDKEGVEE RLLESPGKYC CTVAVR-GNA DTTLVDNVTL  886
NCLIV_039640             ---------- ---------- ---------- ---------- -----R-ANP ----------  392
TGME49_265870            SSYKRGGHLK GELHANGSSR SSSPADTADM ILLDS-GRYC LTVAVR-GNP LTTVVDSVFL  551
HHA_265870               SSYKRGGHLK GELHANGSSH SSSPADTADM ILLDS-GRYC LTVAVR-GNP LTTVVDSVVL  551
XP_002768075.P. marinus  ---------- ---------- ---------- ------EPVL VAIAVKYGGA NLRLIDNMWM  325
XP_002767048.P. marinus  ---------- ---------- ---------- ------EPVL VAIAVKYGGA NLRLIDNMWM  325
sp|P31663|PANC_ECOLI     ---------- ---------- ---------- ------KRAV ILVAAW-LGD -ARLIDNKMV  280
sp|P9WIL5|PANC_MYCTU     ---------- ---------- ---------- ---------- LLVAAR-LGT -TRLLDNIAI  285

                                 1390       1400       1410       1420       1430       1440
                         ....|....| ....|....| ....|....| ....|....| ....|....| ....|....|
ETH_00031750             SDTHKFGDLL PPVPNSAEAV PSAAAALLPL HAAPCVLLLR SLSLQLLQKS PPAAAAAAAA  455
Vbra_16581               EAPKG-DSAQ QRQQQSGWMA SWLGAGK--- ---------- ---------- ----------  988
Cvel_3471                RVP------- ---------- ---------- ---------- ---------- ----------  1132
SN3_00101300             TDQPQCGDLL PPPLNVPWGR NSAAAKHSFF ---GSFLVLA SLGMHLRYLN EDDVDLLLQA  943
NCLIV_039640             ---------- ---------- ---------- ---------- ---------- ----------  392
TGME49_265870            IPNNR-EDLL PPLFNTPWDH HCVEPKYACL ---PSFLVFS SFGFHLRPLR PSDLKLVAAA  607
HHA_265870               IPNNR-EDLL PPLFNTPWDH HCVEPKYACL ---PSFLLFS SFGFHLRPLR PSDLKLVVAA  607
XP_002768075.P. marinus  DNQKHA---- ---------- ---------- ---------- ---------- ----------  331
XP_002767048.P. marinus  DNQKHA---- ---------- ---------- ---------- ---------- ----------  331
sp|P31663|PANC_ECOLI     ELA------- ---------- ---------- ---------- ---------- ----------  283
sp|P9WIL5|PANC_MYCTU     EIGTFAGTDR PDGYRAILES HWRN------ ---------- ---------- ----------  309

                                 1450       1460       1470       1480       1490       1500
                         ....|....| ....|....| ....|....| ....|....| ....|....| ....|....|
ETH_00031750             AEWAQTVEE- ---------- ---------- ---------- ---------- ----------  464
Vbra_16581               ---------- ---------- ---------- ---------- ---------- ----------  988
Cvel_3471                ---------- ---------- ---------- ---------- ---------- ----------  1132
SN3_00101300             EEEDMEPRQN GPRLLLPPLS PAVGATAACQ RQTEGEKNLG CSTRQQPVAA AGPGTETREP  1003
NCLIV_039640             ---------- ---------- ---------- ---------- ---------- ----------  392
TGME49_265870            EQEDARVES- ---------- ---------- ---------- ---------- ----------  616
HHA_265870               EQEDARVES- ---------- ---------- ---------- ---------- ----------  616
XP_002768075.P. marinus  ---------- ---------- ---------- ---------- ---------- ----------  331
XP_002767048.P. marinus  ---------- ---------- ---------- ---------- ---------- ----------  331
sp|P31663|PANC_ECOLI     ---------- ---------- ---------- ---------- ---------- ----------  283
sp|P9WIL5|PANC_MYCTU     ---------- ---------- ---------- ---------- ---------- ----------  309

                                 1510       1520       1530       1540       1550       1560
                         ....|....| ....|....| ....|....| ....|....| ....|....| ....|....|
ETH_00031750             ---------- ---------- ---------- ---------- --------LE ALELSDPVVS  476
Vbra_16581               ---------- ---------- ---------- ---------- ---------- ----------  988
Cvel_3471                ---------- ---------- ---------- ---------- ---------- ----------  1132
SN3_00101300             LLQQQQRIGV CEGTRARTGE RMHILRWLQQ QRRTLFEVEA AGTRKTLWMK KQGDTAAAGA  1063
NCLIV_039640             ---------- ---------- ---------- ---------- ---------- ----------  392
TGME49_265870            ---------- ---------- ---------- ---------- ---------Q QSAKSAGVAA  627
HHA_265870               ---------- ---------- ---------- ---------- ---------Q QSAKSTGADA  627
XP_002768075.P. marinus  ---------- ---------- ---------- ---------- ---------- ----------  331
XP_002767048.P. marinus  ---------- ---------- ---------- ---------- ---------- ----------  331
sp|P31663|PANC_ECOLI     ---------- ---------- ---------- ---------- ---------- ----------  283
sp|P9WIL5|PANC_MYCTU     ---------- ---------- ---------- ---------- ---------- ----------  309

                                 1570       1580       1590       1600       1610       1620
                         ....|....| ....|....| ....|....| ....|....| ....|....| ....|....|
ETH_00031750             SLSSAERQKA WPWASAAAAA AAAP------ ---------- ---------- ----------  500
Vbra_16581               ---------- ---------- ---------- ---------- ---------- ----------  988
Cvel_3471                ---------- ---------- ---------- ---------- ---------- ----------  1132
SN3_00101300             AEQSCVRPCA QQSSQVHSPA TSGSGSTFQN SSNVCRVRAV GLFKYDLMDE CSSSSNETAR  1123
NCLIV_039640             ---------- ---------- ---------- ---------- ---------- ----------  392
TGME49_265870            PETCCTRTFT WMHAGALSPT STHD------ ------VWAL GLFKRRL--- ----------  662
HHA_265870               PETCCTRTFT WMHAGALSPT STHD------ ------VWAL GLFKRRL--- ----------  662
XP_002768075.P. marinus  ---------- ---------- ---------- ---------- ---------- ----------  331
XP_002767048.P. marinus  ---------- ---------- ---------- ---------- ---------- ----------  331
sp|P31663|PANC_ECOLI     ---------- ---------- ---------- ---------- ---------- ----------  283
sp|P9WIL5|PANC_MYCTU     ---------- ---------- ---------- ---------- ---------- ----------  309

                                 1630       1640       1650       1660       1670       1680
```

```
                           ....|....| ....|....| ....|....| ....|....| ....|....| ....|....|
ETH_00031750               ---------- ---------- ---------- ---------- ---------- ----------  500
Vbra_16581                 ---------- ---------- ---------- ---------- ---------- ----------  988
Cvel_3471                  ---------- ---------- ---------- ---------- ---------- ----------  1132
SN3_00101300               LTSHCSDRAA LEADRISVAS RGGADASIQS KDTRRREGRH VAAVGDTSAN RTALHSGGRT  1183
NCLIV_039640               ---------- ---------- ---------- ---------- ---------- ----------  392
TGME49_265870              ---------- ---------- ---------- ---------- -----DTARN TVDFSSDTQV  677
HHA_265870                 ---------- ---------- ---------- ---------- -----DSARN TVDVSSDTQV  677
XP_002768075.P. marinus    ---------- ---------- ---------- ---------- ---------- ----------  331
XP_002767048.P. marinus    ---------- ---------- ---------- ---------- ---------- ----------  331
sp|P31663|PANC_ECOLI       ---------- ---------- ---------- ---------- ---------- ----------  283
sp|P9WIL5|PANC_MYCTU       ---------- ---------- ---------- ---------- ---------- ----------  309

                                 1690       1700       1710       1720       1730       1740
                           ....|....| ....|....| ....|....| ....|....| ....|....| ....|....|
ETH_00031750               ---------- ---------- ---------- ---------- --SSYFLLRR LRCTDSPGEL  518
Vbra_16581                 ---------- ---------- ---------- ---------- ---------- ----------  988
Cvel_3471                  ---------- ---------- ---------- ---------- ---------- ----------  1132
SN3_00101300               PAERDTCLSG AADRCSDSGN PRETTAKSGR FKRRERLIGY VWAEEYAGEE QGGPPQKRER  1243
NCLIV_039640               ---------- ---------- ---------- ---------- ---------- ----------  392
TGME49_265870              PDGNEV---- ---------- ---------- ------LIGY IWAD------ --------AE  693
HHA_265870                 PDGNEV---- ---------- ---------- ------LIGY IWAD------ --------AE  693
XP_002768075.P. marinus    ---------- ---------- ---------- ---------- ---------- ----------  331
XP_002767048.P. marinus    ---------- ---------- ---------- ---------- ---------- ----------  331
sp|P31663|PANC_ECOLI       ---------- ---------- ---------- ---------- ---------- ----------  283
sp|P9WIL5|PANC_MYCTU       ---------- ---------- ---------- ---------- ---------- ----------  309

                                 1750       1760       1770       1780       1790       1800
                           ....|....| ....|....| ....|....| ....|....| ....|....| ....|....|
ETH_00031750               VAYLAFTRVQ NKSICIQRIF VAPKERRKGI AEAFFCTCML LLQQQLLQQQ LQQQQASNEQ  578
Vbra_16581                 ---------- ---------- ---------- ---------- ---------- ----------  988
Cvel_3471                  ---------- ---------- ---------- ---------- ---------- ----------  1132
SN3_00101300               KEGCSASGNV CRVLRVNRVF LKRERRRQTL GTHLLRAFIA ATYKQLLEEQ QQAVSVAPRH  1303
NCLIV_039640               ---------- ---------- ---------- ---------- ---------- ----------  392
TGME49_265870              TPRAAAAAGG ASPWRLRRGF ITRARRRRTF GTHLLKAFLA TLYKEKVSSS RTA-------  746
HHA_265870                 TPHAAAAAGG ASPWRLRRGF ITRARRRRTF GTHLLKAFLA TLYREKVSSN RTA-------  746
XP_002768075.P. marinus    ---------- ---------- ---------- ---------- ---------- ----------  331
XP_002767048.P. marinus    ---------- ---------- ---------- ---------- ---------- ----------  331
sp|P31663|PANC_ECOLI       ---------- ---------- ---------- ---------- ---------- ----------  283
sp|P9WIL5|PANC_MYCTU       ---------- ---------- ---------- ---------- ---------- ----------  309

                                 1810       1820       1830       1840       1850       1860
                           ....|....| ....|....| ....|....| ....|....| ....|....| ....|....|
ETH_00031750               ---------- ---------- ---------- ---------- ---------- ----------  578
Vbra_16581                 ---------- ---------- ---------- ---------- ---------- ----------  988
Cvel_3471                  ---------- ---------- ---------- ---------- ---------- ----------  1132
SN3_00101300               HRPPSAPVLH QRQQQEAVLK TAGHTGPTVK GRPQECEDRE AVVCKIRRTE EEQEMQEDAI  1363
NCLIV_039640               ---------- ---------- ---------- ---------- ---------- ----------  392
TGME49_265870              ---------- ---------- ---------- ---------- ---------- ----------  746
HHA_265870                 ---------- ---------- ---------- ---------- ---------- ----------  746
XP_002768075.P. marinus    ---------- ---------- ---------- ---------- ---------- ----------  331
XP_002767048.P. marinus    ---------- ---------- ---------- ---------- ---------- ----------  331
sp|P31663|PANC_ECOLI       ---------- ---------- ---------- ---------- ---------- ----------  283
sp|P9WIL5|PANC_MYCTU       ---------- ---------- ---------- ---------- ---------- ----------  309

                                 1870       1880       1890       1900       1910       1920
                           ....|....| ....|....| ....|....| ....|....| ....|....| ....|....|
ETH_00031750               ---------- ---------- ---------- ---------- ---------- ---QVTCVVP  585
Vbra_16581                 ---------- ---------- ---------- ---------- ---------- ----------  988
Cvel_3471                  ---------- ---------- ---------- ---------- ---------- ----------  1132
SN3_00101300               TEATETRKSR ERGKLETLCV EGETESTLEG GATEGKEWKK SKHEMEGEIS CTVQLENVPH  1423
NCLIV_039640               ---------- ---------- ---------- ---------- ---------- ----------  392
TGME49_265870              --------SR EV-------- ---------- ---------- ---------- ---QGDPMPA  757
HHA_265870                 --------SR EV-------- ---------- ---------- ---------- ---QGDPMPA  757
XP_002768075.P. marinus    ---------- ---------- ---------- ---------- ---------- ----------  331
XP_002767048.P. marinus    ---------- ---------- ---------- ---------- ---------- ----------  331
sp|P31663|PANC_ECOLI       ---------- ---------- ---------- ---------- ---------- ----------  283
sp|P9WIL5|PANC_MYCTU       ---------- ---------- ---------- ---------- ---------- ----------  309

                                 1930       1940       1950       1960       1970
                           ....|....| ....|....| ....|....| ....|....| ....|....| ...
ETH_00031750               QLALKVMEKS FGFTPEGGPH EGGPHQGGPH EGGTPTVAVS LELNALAQRL LRP 638
Vbra_16581                 ---------- ---------- ---------- ---------- ---------- --- 988
Cvel_3471                  ---------- ---------- ---------- ---------- ---------- --- 1132
SN3_00101300               YLVGVLREGG FCVDTAAAET TRAQTYKGSC CCMHVSLAQW YNAFVLNANV --- 1473
NCLIV_039640               ---------- ---------- ---------- ---------- ---------- --- 392
TGME49_265870              WLAPAVSECG FSL------- LDSTNLSSGE CRLHLNLDKW FRTYVTDLRL --- 800
```

```
HHA_265870                WLAPAVSECG FSL------- MESTNLSSGE CRLHLNLDKW FRANVTDLGL --- 800
XP_002768075.P. marinus   ---------- ---------- ---------- ---------- ---------- --- 331
XP_002767048.P. marinus   ---------- ---------- ---------- ---------- ---------- --- 331
sp|P31663|PANC_ECOLI      ---------- ---------- ---------- ---------- ---------- --- 283
sp|P9WIL5|PANC_MYCTU      ---------- ---------- ---------- ---------- ---------- --- 309
```
