# Supplementary material for: Pantothenate biosynthesis is critical for chronic infection by the neurotropic parasite Toxoplasma gondii
Source: Nat Commun. 2022 Jan 17;13:345. doi: 10.1038/s41467-022-27996-4 (PMC8764084; doi:10.1038/s41467-022-27996-4)
Supplement: Supplementary file 6 — Reporting Summary [file 41467_2022_27996_MOESM6_ESM.pdf]

nature research

Corresponding author(s): Dominique Soldati-Favre

Last updated by author(s): Dec 15, 2021

Reporting Summary

Nature Research wishes to improve the reproducibility of the work that we publish. This form provides structure for consistency and transparency in reporting. For further information on Nature Research policies, see our Editorial Policies and the Editorial Policy Checklist.

Statistics

For all statistical analyses, confirm that the following items are present in the figure legend, table legend, main text, or Methods section.

n/a	Confirmed	
☐	☒	The exact sample size (*n*) for each experimental group/condition, given as a discrete number and unit of measurement
☐	☒	A statement on whether measurements were taken from distinct samples or whether the same sample was measured repeatedly
☐	☒	The statistical test(s) used AND whether they are one- or two-sided<br>*Only common tests should be described solely by name; describe more complex techniques in the Methods section.*
☒	☐	A description of all covariates tested
☐	☒	A description of any assumptions or corrections, such as tests of normality and adjustment for multiple comparisons
☐	☒	A full description of the statistical parameters including central tendency (e.g. means) or other basic estimates (e.g. regression coefficient) AND variation (e.g. standard deviation) or associated estimates of uncertainty (e.g. confidence intervals)
☐	☒	For null hypothesis testing, the test statistic (e.g. *F*, *t*, *r*) with confidence intervals, effect sizes, degrees of freedom and *P* value noted<br>*Give P values as exact values whenever suitable.*
☒	☐	For Bayesian analysis, information on the choice of priors and Markov chain Monte Carlo settings
☒	☐	For hierarchical and complex designs, identification of the appropriate level for tests and full reporting of outcomes
☒	☐	Estimates of effect sizes (e.g. Cohen's *d*, Pearson's *r*), indicating how they were calculated

Our web collection on statistics for biologists contains articles on many of the points above.

Software and code

Policy information about availability of computer code

Data collection	Biorad ImageLab 6.0; Xcalibur v.4.2 (Thermo); Zeiss AxioVision 4.9.1
Data analysis	Excel 2016 (Microsoft); GraphPad Prism 7/9; ImageJ 1.53; Xcalibur v.4.2 (Thermo); Skyline 21.1.0.146; OpenChrom Community Edition 1.1.0 (Diels); El Maven 0.12.0 (Elucidata); Biorad ImageLab 6.0

For manuscripts utilizing custom algorithms or software that are central to the research but not yet described in published literature, software must be made available to editors and reviewers. We strongly encourage code deposition in a community repository (e.g. GitHub). See the Nature Research guidelines for submitting code & software for further information.

Data

Policy information about availability of data

All manuscripts must include a data availability statement. This statement should provide the following information, where applicable:

- Accession codes, unique identifiers, or web links for publicly available datasets
- A list of figures that have associated raw data
- A description of any restrictions on data availability

The authors declare that the data supporting the findings of this study are available within the paper and its supplementary information files. Source data are provided with this paper. RNA sequencing data is deposited on the European Nucleotide Archive (ENA) following the ENA guidelines. The files can be accessed and downloaded via the link: http://www.ebi.ac.uk/ena/data/view/PRJEB43225.
RNA analysis output: http://dx.doi.org/10.17632/zyrz6dcc85.1
Metabolomics datasets can be found in the Yareta (Geneva) repository following the link:
https://doi.org/10.26037/yareta:bvz6yrckafdrxmzgn5hpuumkue.

Field-specific reporting

Please select the one below that is the best fit for your research. If you are not sure, read the appropriate sections before making your selection.

- [x] Life sciences
- [ ] Behavioural & social sciences
- [ ] Ecological, evolutionary & environmental sciences

For a reference copy of the document with all sections, see nature.com/documents/nr-reporting-summary-flat.pdf

Life sciences study design

All studies must disclose on these points even when the disclosure is negative.

Sample size	Sample size were chosen based on published literature for the performed experiments [1-2]. All experiments were performed with enough biological replicates (a minimum of 3) to allow relevant statistical analysis.<br>1. Kloehn J, Oppenheim RD, Siddiqui G, De Bock PJ, Kumar Dogga S, Coute Y, Hakimi MA, Creek DJ, Soldati-Favre D. Multi-omics analysis delineates the distinct functions of sub-cellular acetyl-CoA pools in Toxoplasma gondii. BMC Biol. 2020 Jun 16;18(1):67. doi: 10.1186/s12915-020-00791-7.<br>2. Di Cristina, M., Dou, Z., Lunghi, M. et al. Toxoplasma depends on lysosomal consumption of autophagosomes for persistent infection. Nat Microbiol 2, 17096 (2017). https://doi.org/10.1038/nmicrobiol.2017.96
Data exclusions	No data was excluded from the presented findings
Replication	All presented findings were replicable. Number of replicates (minimum 3 per experiment) are indicated in the figure legends
Randomization	For LC-MS analyses, samples were analyzed in a randomized order to avoid batch effects. Experimental groups for in vivo infection were randomized, as well as their sample analysis. Immunofluorescences for quantification were randomized. No randomization was performed or was necessary for cell culture or other experiments.
Blinding	Blinding was performed for quantification of IFA experiments. In vivo data was blinded and confirmed independently by 2 authors. For all other experiments no blinding was possible or necessary. This was due to the qualitative, rather than quantitative nature of the experiment (eg western blot)

Reporting for specific materials, systems and methods

We require information from authors about some types of materials, experimental systems and methods used in many studies. Here, indicate whether each material, system or method listed is relevant to your study. If you are not sure if a list item applies to your research, read the appropriate section before selecting a response.

Materials & experimental systems

n/a	Involved in the study	
☐	☒	Antibodies
☐	☒	Eukaryotic cell lines
☒	☐	Palaeontology and archaeology
☐	☒	Animals and other organisms
☒	☐	Human research participants
☒	☐	Clinical data
☒	☐	Dual use research of concern

Methods

n/a	Involved in the study	
☒	☐	ChIP-seq
☒	☐	Flow cytometry
☒	☐	MRI-based neuroimaging

Antibodies

Antibodies used	Polyclonal rabbit anti GAP45 (1:10,000), anti HA (1:1,000, Sigma H6908), anti HSP70 (1:1,000), anti CPN60 (1:3,000), anti-catalase (1:2,000), anti IMC1 (1:2,000). Monoclonal mouse anti actin (1:20), anti GRA1 and anti GRA3 (1:20, gifts of Dr. J. F. Dubremetz), anti myc (1:10, 9E10), anti Ty (1:10, BB2), anti P21 (1:10), anti SAG1 (1:10, gift of Dr. J. F. Dubremetz). FITC conjugated lectin (DBA, 1:500, Vector Laboratories FL-1031-2). Secondary antibodies for immunofluorescence: anti mouse Alexa fluor 405 (1:3000, Invitrogen A31553), 488 (1:3000, Invitrogen A11001), 594 (1:3000, Invitrogen A11005), anti-rabbit Alexa fluor 488 (1:3000, Invitrogen A11008), 594 (1:3000, Invitrogen A11012). Secondary antibodies for western blot: anti mouse HRP (1:3000, Sigma A5278), anti-rabbit HRP (1:3000, Sigma A8275). All antibody used are mentioned and referenced in the methods section.
Validation	All antibodies used were validated in prior publications, and are described in the reference. Validation was performed with parasite knock-out lines, or demonstration of co-localization with known markers of parasite compartments.

Eukaryotic cell lines

Policy information about cell lines

Cell line source(s)	Human Foreskin Fibroblasts: ATCC SCRC-1041, Sf9 insect cells: ATCC Sf9 CRL-1711
Authentication	No authentication for either cell line
Mycoplasma contamination	All cell lines tested negative for Mycoplasma
Commonly misidentified lines (See ICLAC register)	No commonly misidentified cell lines were used in the study

Animals and other organisms

Policy information about studies involving animals; ARRIVE guidelines recommended for reporting animal research

Laboratory animals	The study involved 7 week old female mice of the strains CD1 and B6CBAf1
Wild animals	No wild animals were used in the study
Field-collected samples	No field-collected samples were used in the study
Ethics oversight	All animal experiments were conducted with the authorization numbers GE150-16 and GE121-19, according to the guidelines and regulations issued by the Swiss Federal Veterinary Office. No human samples were used for these experiments.

Note that full information on the approval of the study protocol must also be provided in the manuscript.
